# Global, regional, and national health inequalities of Alzheimer’s disease and Parkinson’s disease in 204 countries, 1990–2019

**DOI:** 10.1186/s12939-024-02212-5

**Published:** 2024-06-19

**Authors:** Zixiang Ji, Qi Chen, Jing Yang, Jiazhe Hou, Hengjing Wu, Lijuan Zhang

**Affiliations:** 1 Clinical Center for Intelligent Rehabilitation Research, Shanghai YangZhi Rehabilitation Hospital Tongji University School of Medicine, Tongji University, Shanghai, 201619 China; 2 Department of Epidemiology, School of Public Health, Tongji University School of Medicine, Tongji University, 2209 Xingguang Road, Songjiang, Shanghai, 201619 China; 3 School of Public Health, Nanjing Medical University, Nanjing, Jiangsu province 211166 China

**Keywords:** “Alzheimer Disease/epidemiology“[Mesh], “Parkinson Disease/epidemiology“[Mesh], “Health Inequities“[Mesh], “Global Burden of Disease/trends“[Mesh], “Incidence/trends“[Mesh], “Prevalence/trends“[Mesh], “Disability-Adjusted Life Years /trends“[Mesh]

## Abstract

**Background:**

Alzheimer’s disease and related dementias (ADRD) and Parkinson’s disease (PD), pose growing global health challenges. Socio-demographic and economic development acts paradoxically, complicating the process that determines how governments worldwide designate policies and allocate resources for healthcare.

**Methods:**

We extracted data on ADRD and PD in 204 countries from the Global Burden of Disease 2019 database. Health disparities were estimated using the slope index of inequality (SII), and concentration index (CIX) based on the socio-demographic index. Estimated annual percentage changes (EAPCs) were employed to evaluate temporal trends.

**Results:**

Globally, the SII increased from 255.4 [95% confidence interval (CI), 215.2 to 295.5)] in 1990 to 559.3 (95% CI, 497.2 to 621.3) in 2019 for ADRD, and grew from 66.0 (95% CI, 54.9 to 77.2) in 1990 to 132.5 (95% CI, 118.1 to 147.0) in 2019 for PD; CIX rose from 33.7 (95% CI, 25.8 to 41.6) in 1990 to 36.9 (95% CI, 27.8 to 46.1) in 2019 for ADRD, and expanded from 22.2 (95% CI, 21.3 to 23.0) in 1990 to 29.0 (95% CI, 27.8 to 30.3) in 2019 for PD. Age-standardized disability-adjusted life years displayed considerable upward trends for ADRD [EAPC = 0.43 (95% CI, 0.27 to 0.59)] and PD [0.34 (95% CI, 0.29 to 0.38)].

**Conclusions:**

Globally, the burden of ADRD and PD continues to increase with growing health disparities. Variations in health inequalities and the impact of socioeconomic development on disease trends underscored the need for targeted policies and strategies, with heightened awareness, preventive measures, and active management of risk factors.

**Supplementary Information:**

The online version contains supplementary material available at 10.1186/s12939-024-02212-5.

## Background

Neurodegeneration is characterized by a progressive loss of neuronal function and structure to bring enormous impairment to cognitive-motor function. The underlying main histopathological hallmark involves the aggregation of pathologic proteins including beta-amyloid deposits and hyperphosphorylated tau proteins, inflammation, and neuronal cell death [1, 2], and all these contribute to a variety of issues consisting of memory loss, attention problems, slowed information processing, motor skill loss [1]. Alzheimer’s disease and related dementias (ADRD) and Parkinson’s disease (PD) serve as the two representations of degenerative neurologic disorders [3]. Approximately 60 million individuals worldwide struggled with ADRD and PD in 2019 [4, 5], and the burden of diseases has been on the rise over the years [4, 6–9] with age [10], unhealthy lifestyles [6, 11], and poorly understood genetic and environmental factors [12, 13].

It is commonly perceived that higher disease burdens are often directly associated with worse socio-economic status and fewer healthcare resources [14–16]. However, as a consequence of socio-economic progress, burdens of some disorders, such as falls among the elderly, mental health issues among youngsters, and neurological disorders, have tended to grow recently [5, 17, 18], which seem to conflict with health and well-being within the context of sociodemographic and economic development. Among these, mental illnesses, Alzheimer’s disease, and Parkinson’s disease, etc., are more directly associated with societal changes, which seem to stem from the growth of an aging population [10], rising social stress [19, 20], deteriorating sleep issues [21], and illogical eating habits [22]. With the rapid economic growth, the world has been experiencing changes in people´s socioeconomic statuses, lifestyles, and societal pressure [19, 20, 22]. The rapid development of society also imposes the pressure of a high-speed economic life, which originates from superiors, peers and even younger generations. The accelerated pace of life also changes the pace of life, which is reflected in the irregularity of the daily diet (number and types of meals, etc.) [22], poorer quality of sleep and less time [21], increasing the social burden of neuropsychiatric disorders, especially in ADRD and PD. Diverse country-level health disparities for various diseases in multiple categories (e.g., sex) appear to offer a valuable research foundation for determining causality concerning some factors that emerge alongside economic advancements and to which different populations are exposed distinctly. These tendencies appear to notify medical professionals about the fact that economic progress shouldn’t be always beneficial, with the necessity of giving certain elements’ emergence more consideration in light of the overall trend of economic growth.

Data on the global burden of disease or injury provide comprehensive and essential information for subsequent studies in ADRD and PD. The aforementioned information was employed in this research to evaluate cross-national health inequality indicators based on socio-demographic index (SDI) levels, a standard health inequality analysis methodology recommended by the World Health Organization (WHO), as well as to determine the magnitude and trends over time. With the implementation of Estimated Annual Percentage Changes (EAPCs), the degree of correlation between trend changes in disease and SDI and age-standardized rates (ASRs) was assessed as well. We aimed to provide a scientific foundation for the development of relevant policies and strategies and the distribution of health resources in real-world settings.

## Methods

### Data source

Data were derived from the Global Burden of Disease 2019 (GBD 2019), conducted by the Institute for Health Metrics and Evaluation (IHME) [4, 23] using the Global Health Data Exchange (GHDx) online tool [24–26] (http://ghdx.healthdata.org/gbd-results-tool). In the present study, annual cases and ASRs with uncertainty intervals (UIs) for incidence (ASIRs), deaths (ASDRs), and disability-adjusted life years (ASRs of DALYs) of ADRD and PD from 1990 to 2019 were extracted. The data were categorized into the following different subgroups including sex (whole population, male, female), age (0–4, 5–9, 10–14, …, 90–94, 95+), and location (204 countries and 21 regions). Meanwhile, the SDI was also employed to measure the sociodemographic development level of a nation or territory based on characteristics such as economics, education levels, and fertility rate [24, 27, 28] (eMethods in the Supplement, https://ghdx.healthdata.org/record/ihme-data/gbd-2019-socio-demographic-index-sdi-1950-2019).

### National inequality analysis

The Absolute Index of Inequality (AI) and the Relative of Inequality Index (RI) are a pair of fundamental indices implemented in epidemiological investigations to assess socioeconomic health disparities [29]. For structured categories, the complex inequality measurements slope index of inequality (SII) (simply interpreted as the occurrence of events in the highest-SDI regions and the lowest-SDI regions, in this case, the relative ratio of the burden of disease.) and concentration index (CIX) (simply interpreted as the occurrence of events in the highest-SDI regions and the lowest-SDI regions, in this case, the direct difference of the burden of disease.) are respectively consulted to measure absolute and relative inequalities [29]. The SII was calculated by regressing country-level crude DALY rates owing to ADRD and PD in all-age groups on an SDI-related relative social position scale, defined as the midpoint of the cumulative class interval of the population ranked by SDI in 204 countries [30]. The relative social position value was directly implemented to allow for robust linearity to exhibit the disparities between the highest-SDI regions and the lowest-SDI ones [30]. The CIX was calculated by fitting a Lorenz concentration curve to the cumulative relative distributions of the population ranked by SDI and DALYs burden of diseases, then mathematically integrating the area under the curve [30, 31], which also represents the SDI-based health inequalities, highlighting the apparent differences in disease burden.

### Statistical analysis

The EAPCs are measures of annual percentage change that may be utilized to assess the extent of the alteration in a variable over time [32, 33], using the following model: ln (val) = b × year + a, where val is the value for age-standardized rates, b is the coefficient of the year, a is the intercept and year is the calendar year. For this study, the EAPCs and their 95% confidence intervals (95% CIs) of ASIRs, ASDRs, and ASRs of DALYs for AD and PD were calculated to reflect the temporal trends on a linear scale, respectively. When the 95% CIs were computed, if the upper limits equaled less than 0, they exhibited descending tendencies; if the lower limits equaled more than 0, upward trends.

All analyses were based on descriptive epidemiologic methods, and the current emphasis was on variations in ASRs at the SDI level in ADRD and PD, with the differences mainly being global, regional, and national. The Pearson correlation coefficients (ρ index) and *P* values were mostly implemented to quantify the association between ASRs and EAPCs, as well as SDI and EAPCs. Indicators of health and socio-demographics were also combined, as shown by levels and variations of the CIX and SII indicators in accordance with the fluctuations of cross-country SDI. All statistical analyses were completed with R software (Version 4.3.1, MathSoft, Cambridge, MA, US) and it’s considered to be significant when *P* < 0.05 with two-sided tests.

## Results

### Global burden of Alzheimer’s disease and related dementias and Parkinson’s disease

Globally in 2019, 7,236,385 new cases (95% UI, 6,217,239 to 8,232,672) and 1,623,276 deaths (95% UI, 407,465 to 4,205,719) were associated with ADRD, both incidence cases (Ratio _*male vs. female*_=0.59) and death cases (Ratio _*male vs. female*_=0.53) presented higher in females than in males (eTable 2 in Supplement). Total 1,081,723 new cases (95% UI, 953,265 to 1,211,202) and 362,907 deaths (95% UI, 326,855 to 388,200) were observed for PD worldwide in 2019, if 100 new cases and 100 deaths were in females, there were 148 and 135 in males, respectively (eTable 3 in the Supplement). Global ASIRs, ASDRs and ASRs of DALYs displayed upward trends for both AD[EAPCs = 0.31 (95% CI, 0.11 to 0.50) for ASIRs, EAPCs = 0.21 (95% CI, 0.14 to 0.28) for ASDRs], EAPCs = 0.43 (95% CI, 0.27 to 0.59) for ASRs of DALYs] and PD [EAPCs = 0.77 (95% CI, 0.64 to 0.89) for ASIRs; EAPCs = 0.13 (95% CI, 0.08 to 0.19) for ASDRs; EAPCs = 0.34 (95% CI, 0.29 to 0.38) for ASRs of DALYs] (eTable 4 and eTable 5 in the Supplement).

**Fig. 1:**
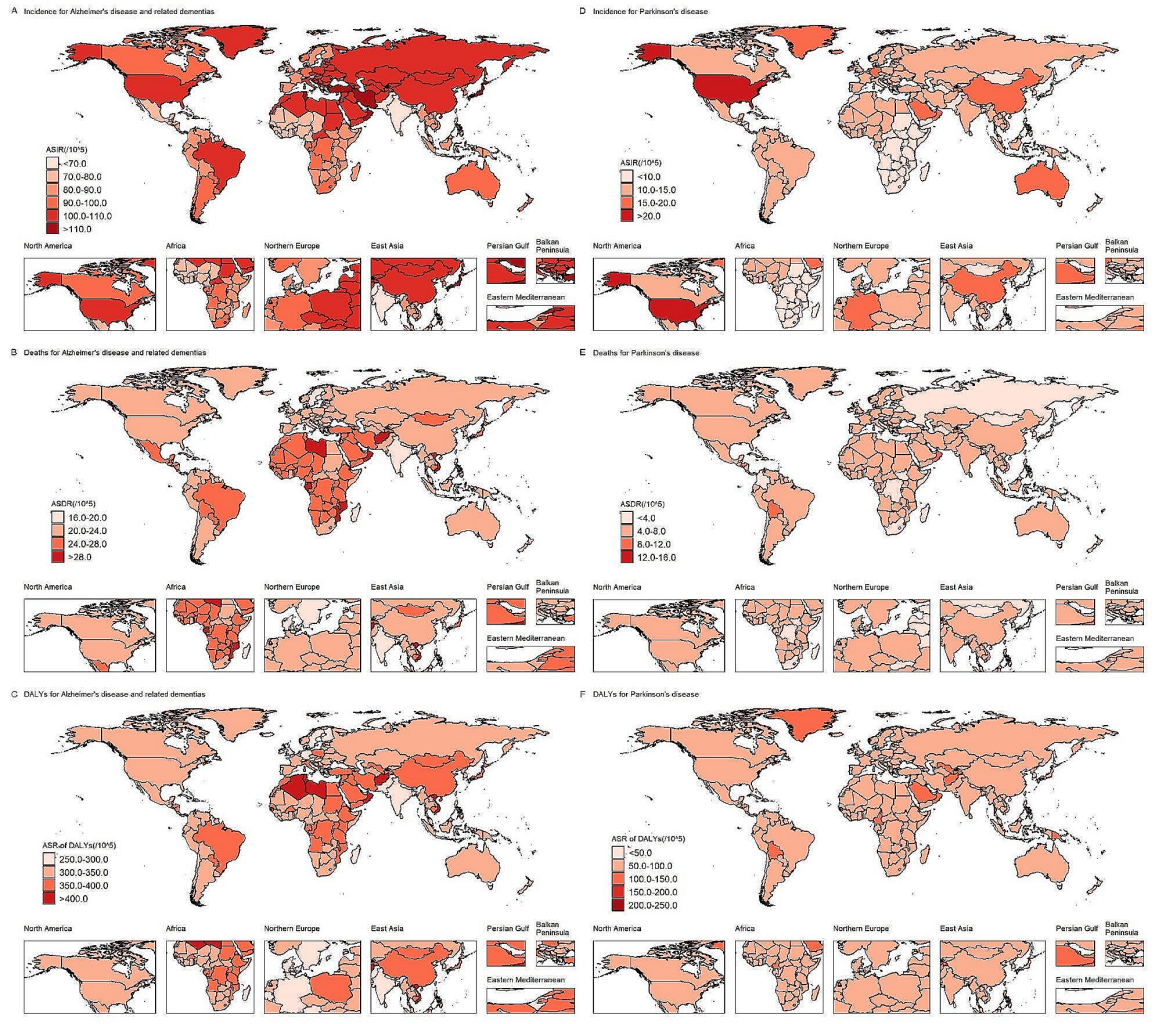
The global maps for age-standardized rates per 100,000 people of incidence, deaths, and DALYs in 2019 for Alzheimer’s disease and related dementias and Parkinson’s disease Abbreviations: DALYs, disability-adjusted life-years; Figures **A**, **B**, and **C** represent age-standardized rates of incidence, deaths, and disability-adjusted life years for Alzheimer’s disease and related dementias, and Figures **D**, **E**, and **F** represent age-standardized rates of incidence, deaths, and disability-adjusted life years for Parkinson’s disease Darker colors in the figures indicate higher age-standardized rates

For AD, at the SDI region level, high-middle SDI region had the most ASIRs [101.68 (95% UI, 86.58 to 116.12)] and age-standardized DALYs rates [348.46 (95% UI, 157.71 to 754.37)] of ADRD, but the most ASDRs occurred in middle SDI region in 2019 (eTable 4 in the Supplement). Across the 21 GBD regions, North Africa and Middle East and high-income Asia Pacific ranked the top two in ASIRs and age-standardized DALYs, but the top two ASDRs occurred in high-income Asia Pacific and Central Sub-Saharan Africa (Fig. 1 and eTable 4 in the Supplement). For PD, high SDI region had the most ASIRs [16.75 (95% UI, 15.11 to 18.31) per 100,000 people], but the most ASDRs [5.28 (95% UI, 4.71 to 5.94)] and age-standardized DALYs [4.92 (95% UI, 4.29 to 5.81)] occurred in low-middle SDI regions (eTable 5 in the Supplement). Across the 21 GBD regions, high-income North America and East Asia ranked the top two in ASIRs, but the top two ASDRs and age-standardized DALYs occurred in Oceania and Western Sub-Saharan Africa (Fig. 1 and eTable 5 in the Supplement).

**Fig. 2:**
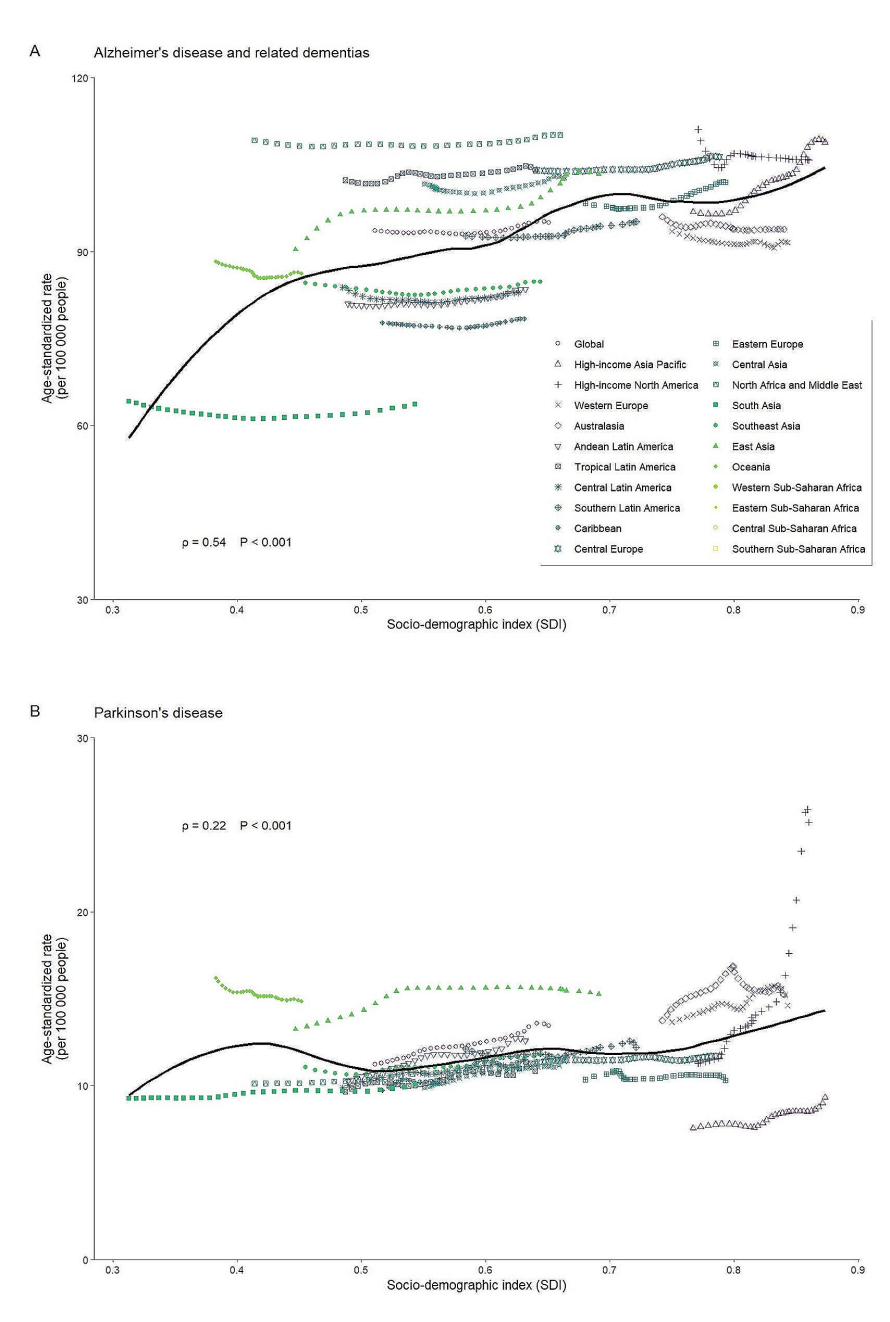
Association between age-standardized incidence rates and socio-demographic index for Alzheimer’s disease and related dementias and Parkinson’s disease The black solid curves coordinate with the overall trend in age-standardized incidence rates, with Pearson correlation coefficients (ρ index) and *P* values indicating the magnitude and statistical significance of the correlation

Upon incorporating worldwide data, it exhibited a significant connection between SDI and ASIRs, indicating regions with higher levels of SDI were at a greater probability of ADRD (*ρ* = 0.54, *P* < 0.001), which was corroborated in PD (*ρ* = 0.22, *P* < 0.001) (Fig. 2). ASIRs of ADRD showed a positive correlation with SDI in 2019 in both males (*ρ* = 0.32, *P* < 0.001) and females (*ρ* = 0.57, *P* < 0.001). For PD, ASIRs showed a positive correlation with SDI in 2019 in males (*ρ* = 0.35, *P* < 0.001), while ASIRs in females showed weak positive correlation with SDI (*ρ* = 0.04, *P* < 0.001) (eFigure 3 in the Supplement).

**Fig. 3:**
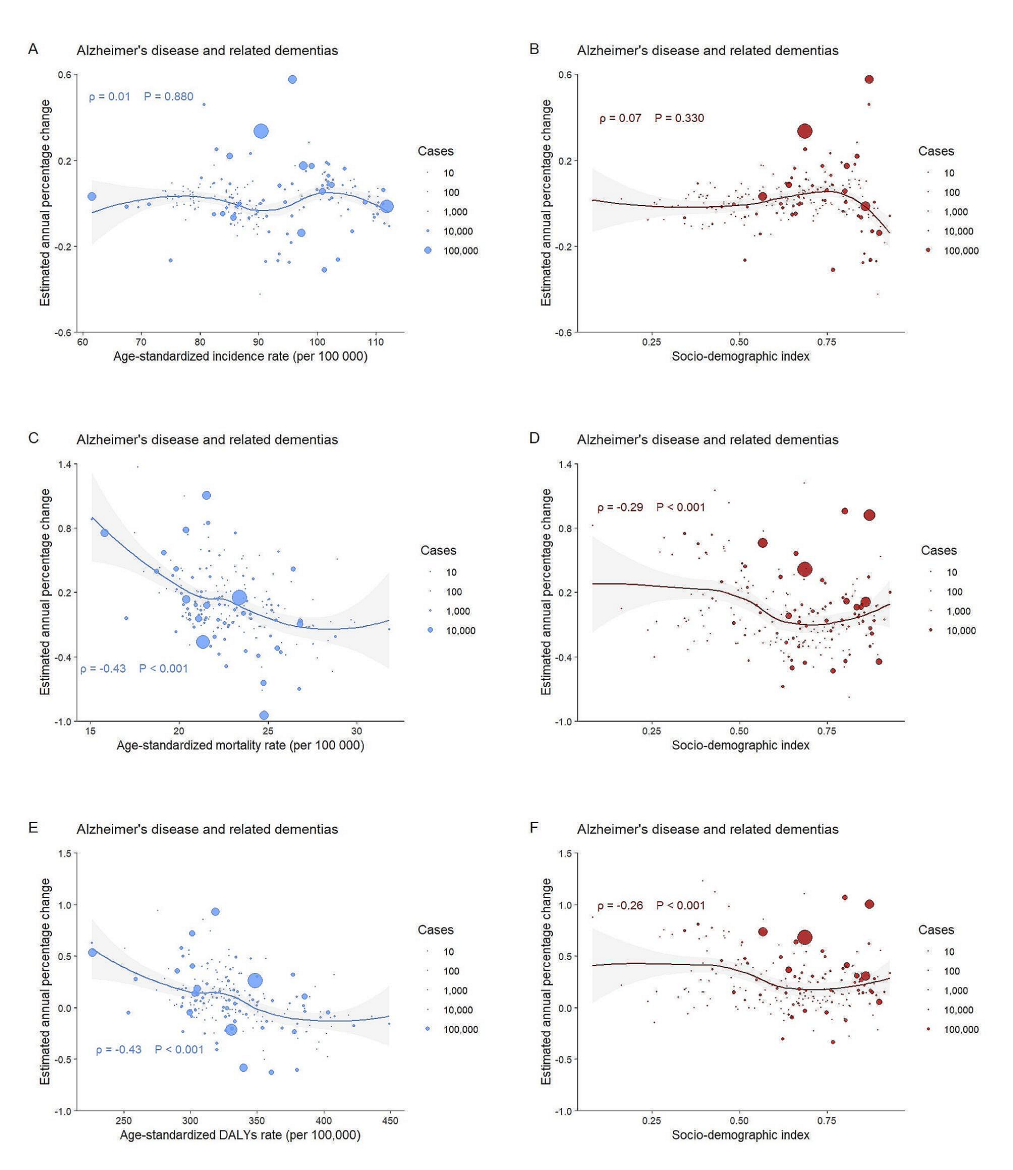
Association between age-standardized rates, socio-demographic index and estimated annual percentage changes, individually, for Alzheimer’s disease and related dementias Circles represent the cases of absolute incidence, deaths, and DALYs, the larger the circle the greater the number of cases. EAPCs are 30-year trends in age-standardized incidence, deaths, and disability-adjusted life year rates per 100 000 people. Pearson correlation coefficients (ρ index) and *P* values indicate the magnitude and statistical significance of the correlation. Figures **A** and **B** denote age-standardized rates, socio-demographic index and estimated annual percentage changes for incidence, individually; Figures **C** and **D** denote age-standardized rates, socio-demographic index and estimated annual percentage changes for deaths, individually; Figures **E** and **F** denote age-standardized rates, socio-demographic index and estimated annual percentage changes for DALYs, individually

**Fig. 4:**
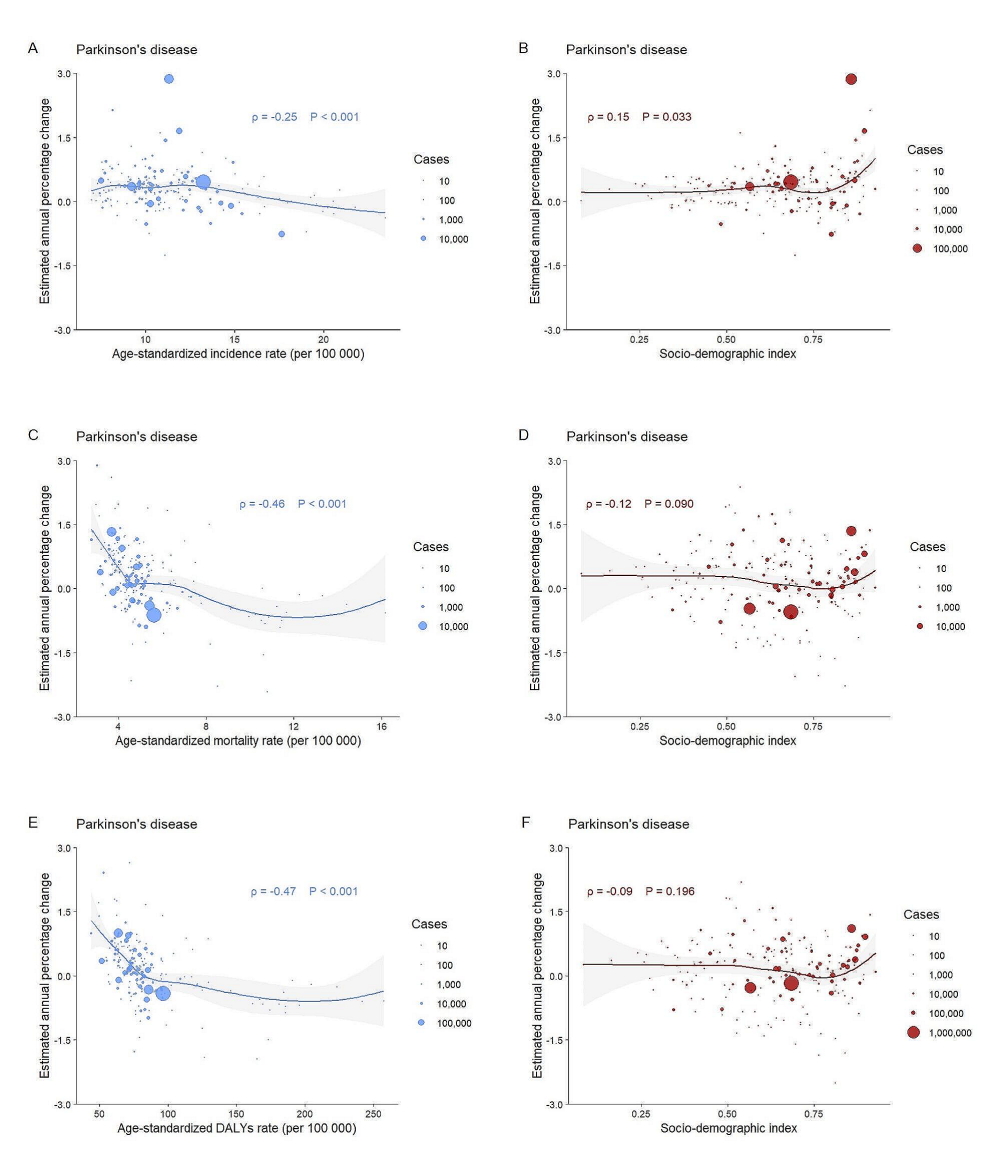
Association between age-standardized incidence rates, socio-demographic index and estimated annual percentage changes, individually, for Parkinson’s disease Circles represent the cases of absolute incidence, deaths, and DALYs, the larger the circle the greater the number of cases. EAPCs are 30-year trends in age-standardized incidence, deaths, and disability-adjusted life year rates per 100 000 people. Pearson correlation coefficients (ρ index) and *P* values indicate the magnitude and statistical significance of the correlation. Figures **A** and **B** denote age-standardized rates, socio-demographic index and estimated annual percentage changes for incidence, individually; Figures **C** and **D** denote age-standardized rates, socio-demographic index and estimated annual percentage changes for deaths, individually; Figures **E** and **F** denote age-standardized rates, socio-demographic index and estimated annual percentage changes for DALYs, individually

EAPCs of incidence did not show a significant correlation with ASIRs or SDI of ADRD from 1990 to 2019; EAPCs of death or DALYs showed a negative correlation with ASDRs or ASR of DALYs (both *ρ* = -0.43), while they showed weak correlations with SDIs (*ρ* = -0.29 for deaths and *ρ* = -0.26 for DALYs, respectively) (Fig. 3). For PD, EAPCs of incidence showed a weak correlation with ASIRs (*ρ* = -0.25, *P* < 0.001) and SDI (*ρ* = 0.15, *P* = 0.033); EAPCs of death or DALYs showed a negative correlation correspondingly with baseline ASDRs or ASR of DALYs in 1990 (both *ρ* more than 0.45), while they did not show significant association with SDIs (both *P* > 0.05) (Fig. 4). These findings were the same across sexes, with the exception that the SDI of PD in females presented a substantial negative connection with EAPCs of DALYs (*ρ* = -0.22, *P* = 0.002) (eFigs. 4, 5 and 6, and 7 in the Supplement).

### National slope index of inequality and concentration index for Alzheimer’s disease and other dementias and Parkinson’s disease

**Fig. 5:**
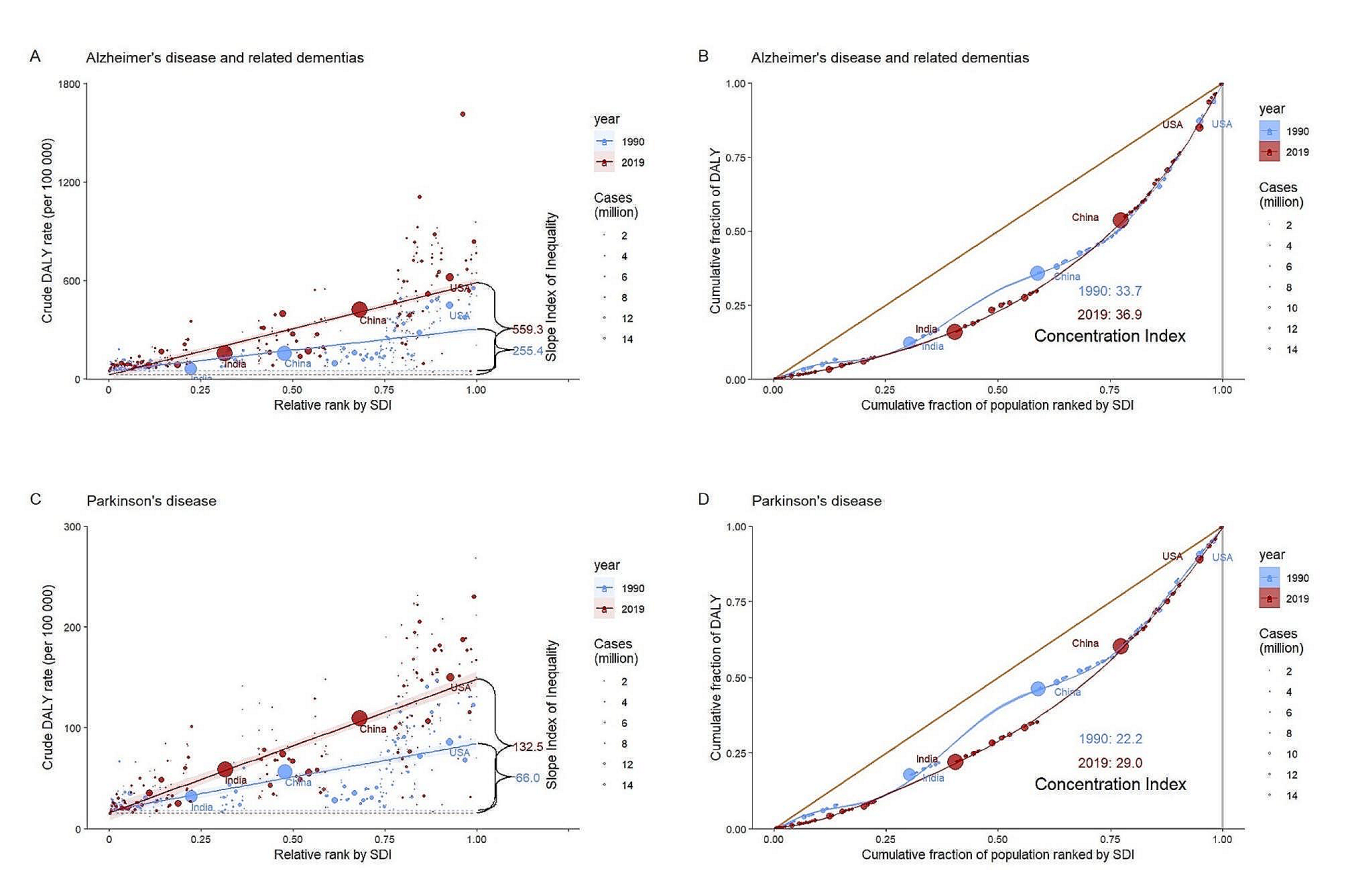
Cross-country slope index of inequality and concentration index in 1990 and 2019 for Alzheimer’s disease and related dementias and Parkinson’s disease among the whole population Circles represent the cases of absolute incidence, deaths, and DALYs, the larger the circle the greater the number of cases. Red lines and circles represent data for 2019, while the blue ones indicate data for 1990. Figures **A** and **C** denote the slope index of inequality for AD and PD, respectively, and Figures **B** and **D** denote the concentration index for AD and PD, respectively

As illustrated by the SII, the gap in the DALYs rate between the highest and lowest SDI countries increased from 255.4 (95% CI, 215.2 to 295.5) in 1990 to 559.3 (95% CI, 497.2 to 621.3) in 2019 for ADRD, and from 66.0 (95% CI, 54.9 to 77.2) in 1990 to 132.5 (95% CI, 118.1 to 147.0) in 2019 for PD; Moreover, CIX showed 33.7 (95% CI, 25.8 to 41.6) in 1990 and 36.9 (95% CI, 27.8 to 46.1) in 2019 for ADRD, 22.2 (95% CI, 21.3 to 23.0) in 1990 and 29.0 (95% CI, 27.8 to 30.3) in 2019 for PD (Fig. 5; Table 1).

**Table 1:** Cross-country slope index of inequality and concentration index according to disability-adjusted life years in 1990 and 2019 for Alzheimer’s disease and related dementias and Parkinson’s disease among both sexes, males, and females

Metric	Disease	Sex	Health inequality index	Year	Estimate	95% CI
Disability-Adjusted Life Years (DALYs)	Alzheimer’s disease and related dementias	Both	Slope index of inequality	1990	255.4	215.2 to 295.5
				2019	559.3	497.2 to 621.3
			Concentration index	1990	33.7	25.8 to 41.6
				2019	36.9	27.8 to 46.1
		Male	Slope index of inequality	1990	157.8	133.1 to 182.6
				2019	387.6	345.5 to 429.7
			Concentration index	1990	26.3	19.7 to 32.8
				2019	32.5	23.8 to 41.1
		Female	Slope index of inequality	1990	342.6	287.2 to 398.1
				2019	724.8	646.7 to 803.0
			Concentration index	1990	37.3	28.6 to 46.0
				2019	39.5	30.1 to 48.9
	Parkinson’s disease	Both	Slope index of inequality	1990	66.0	54.9 to 77.2
				2019	132.5	118.1 to 147.0
			Concentration index	1990	22.2	21.3 to 23.0
				2019	29.0	27.8 to 30.3
		Male	Slope index of inequality	1990	69.1	14.6 to 30.4
				2019	153.4	135.9 to 170.9
			Concentration index	1990	20.2	19.2 to 21.2
				2019	29.8	28.2 to 31.4
		Female	Slope index of inequality	1990	61.8	50.9 to 72.6
				2019	109.1	97.0 to 121.2
			Concentration index	1990	24.6	23.4 to 25.8
				2019	28.1	26.7 to 29.5

Abbreviations: CI: confidence interval

For ADRD, the health inequity disparities between the highest SDI countries and the lowest were larger in females than in males. The SII was 342.6 (95% CI, 287.2 to 398.1) among females and 157.8 (95% CI, 133.1 to 182.6) among males in 1990, respectively, and the SII was 724.8 (95% CI, 646.7 to 803.0) for females whilst 387.6 (95% CI, 345.5 to 429.7) for males in 2019, but the extent of increase in inequity from 1990 to 2019 was greater in males [(387.6-157.8)/157.8 = 145.6%] than in females (111.6%). Inequality rose since 1990 more in males (23.6%) than in females (5.9%), CIX was 37.3 (95% CI, 28.6 to 46.0) in 1990 among females compared 26.3 (95% CI, 19.7 to 32.8) among males, also, the CIX was 39.5 (95% CI, 30.1 to 48.9) in females against 32.5 (95% CI, 23.8 to 41.1) in males in 2019(eFigure 8, eFigure 9 in the Supplement and Table 1).

The discrepancy resided in PD, this disparity was sexually inverted, suggesting males undertook higher health inequalities between the highest SDI levels countries and the lowest than females. In 1990, the SII was 69.1 (95% CI, 14.6 to 30.4) in males and 61.8 (95% CI, 50.9 to 72.6) in females; likewise in 2019, the SII was 153.4 (95% CI, 135.9 to 170.9) for males and 109.1 (95% CI, 97.0 to 121.2) for females; moreover, the degree of expand in inequity from 1990 to 2019 was larger in males [(153.4–69.1)/ 69.1 = 122.0%] than in females (76.5%); simultaneously inequality increased since 1990 more in males (47.5%) than in females (14.2%), CIX was 29.8 (95% CI, 28.2 to 31.4) in males against 28.1 (95% CI, 26.7 to 29.5) in females in 2019; albeit CIX was 24.6 (95% CI, 23.4 to 25.8) in 1990 among females compared 20.2 (95% CI, 19.2 to 21.2) among males (eFigure 8, eFigure 9 in the Supplement and Table 1).

## Discussion

The current longitudinal study concentrated on the SDI-related health inequality indicators, and associations among ASRs, SDI, and EAPCs for ADRD and PD, respectively, two of the most representative degenerative neurological diseases. This particularly statistical analysis (stringing together disease with socio-demographic economics, etc.) remained one of the few ways the area.

When analyzed across the entire population, the absolute and relative inequity indices associated with the SDI-related burden of disease, DALYs for ADRD and PD turned out to be substantially and positively correlated to the SDI level, with nation-states exhibiting greater SDI levels featuring a disproportionately high burden of disease. Both the SII and CIX for ADRD and PD demonstrated increased tendencies from 1990 to 2019, indicating there existed a pattern of expanding disparities in health inequalities among areas parted by SDI levels. This phenomenon appears to emphasize the two-sided traits of sociodemographic economics’ development. Given existing research and experience, regions with high SDI levels tend to possess more social safety and medical care resources [26, 28], facilitating disease prevention, diagnosis, treatment, and healthcare rehabilitation. The genesis of this disparity could be linked to disease risk factors [10, 22, 34–38] such as increasing population aging, lifestyle alternatives, stress, food choices, environment changes, and multiple underlying conditions (stroke, hypertension, atherosclerosis, coronary heart disease, diabetes, depression, etc.). With the development of society, the increase in the proportion of the aging population, environmental pollution caused by deepening industrialization [39, 40], mental disorders brought on the pressure of work competition [41], and cardiovascular and cerebrovascular diseases resulted from the intake of high-oil and high-fat foods [42], would increase the disease burden of degenerative neurological diseases worldwide. To prevent the disease from worsening further and causing related diseases like high blood pressure and psychiatric disorders, it is important for the high-risk groups to receive prevention and treatment as soon as possible. They should also follow their doctors’ instructions for appropriate treatments. High-risk populations should also be more conscious of their lifestyle choices, such as limiting their intake of fried and high-fat red meat.

The health inequity disparities between the highest SDI countries and the lowest for ADRD were larger in females than in males, which was the opposite for PD. Additionally, worldwide ASRs of DALYs for ADRD were larger in females than in males, but the inversed for PD. Sex differences might result from a combination of heredity and environment, where hormones might perform a critical role [43, 44], other factors [22, 45, 46] including work-related stress, lifestyle choices, and pressure management techniques may also contribute to a major impact. Therefore, it is important to incorporate stress-reduction techniques into your life, such as working out, chatting with friends, and other activities.

As SDI levels increased, EAPCs of ASDRs and ASRs of DALYs for ADRD showed considerable downward trends, which was not evident in ASIRs in ADRD as well as ASDRs and ASRs of DALYs in PD, with significant upward tendencies in ASIRs for PD. These indicate that improved healthcare resources are a direct result of greater sociodemographic and economic status [47], which in turn mitigates the increasing trend in ADRD-related fatalities and disease burden, while not yet cases with PD especially in high SDI regions. It is also important to note that the trends (EAPCs) of the AISRs for AD and DALYs and the ASDRs for PD are not substantially correlated with SDI levels, which suggests that the SDI level possesses a minor effect on such aspect and that action should be taken to reduce relevant risk factors for the condition, such as diet, sleep, and the impact of cardiovascular diseases [22, 34, 36–38]. It’s necessary to call for more attention from the government and social sectors to the formulation of policies and the implementation of preventive and therapeutic measures for the disease.

Not to be disregarded, the ASRs for PD and ADRD continue to exhibit notable growing patterns on a worldwide scale, which could be partially related to population aging [10, 48]. From the patient’s perspective, a decline in quality of life results in a decrease in the sense of well-being, and regrettably, this group members tend to grow larger; from the relatives’ perspective, it adds to the burden on the family in terms of the time spent by the caregivers and the financial costs involved; and from the societal and governmental perspectives, it raises the financial outlay and the risk of social instability. The aging process of the population and the high prevalence of related risk diseases (e.g., depression, cardiovascular diseases) may lead to higher ASIRs for ADRD and PD in North Africa, the Middle East, and high-income Asia Pacific, as well as higher ASDRs for PD in high-income North America and East Asia [23, 49]. Higher ASDRs for ADRD and PD in some areas of Sub-Saharan Africa may be the result of inadequate medical attention to health and backwardness in medical treatment (Scarcity of medical workers and medical supplies) [50]. These suggest increased awareness of the modified risk factors for these two disorders among individuals, medical professionals, social organizations, and the government. Together with economic development, other priorities should involve making policies to mitigate the population’s increasing elderly share, protecting and improving the natural environment, learning how to release themselves from stressful positions, scheduling daily meals to prevent and control underlying conditions, and carrying out prevention, diagnosis, treatment, and rehabilitation of underlying diseases actively and timely.

The current study has some strengths. First, this study is one of the few studies which reported effects of health inequalities on burden of neurologic disorders. Second, our study consisted of a global data of 204 counties, and there were no missing data on disease burden and SDI metrics, thereby the results strongly supported the statistical findings. Finally, EAPCs as trending elements to evaluate their correlates with ASRs and SDIs, refining the relationship of sociodemographic indices with disease even further.

Although our study used the latest GBD data to describe the global disease burden attributed to ADRD and PD, there are still several limitations. Firstly, the application of EAPCs has some inherent shortcomings, such as linear assumption (EAPCs assume that the change in the variable is linear, but the change might be nonlinear, leading to an inability of EAPC to capture the true trend), potential bias (EAPCs may be affected by potential biases, such as missing or incomplete data, which can lead to inaccurate results). Secondly, linear regression itself has some limitations, mainly including assumptions of linearity and independence of errors: it assumes that the relationship between the independent and dependent variables is linear, however, the model might produce inaccurate results if this assumption is violated; meanwhile, it assumes that the errors (residuals) are independent of each other, in other words, the error for one observation should not be related to the error for another observation. Thirdly, the data might have heterogeneity because GBD database contains data from all over the world, and disease measures and reports might be diverse in different regions [51], therefore, each region may have more or fewer ADRD or PD cases compared to our estimate. GBD 2019 makes substantial efforts to enhance the comparability of results by applying corrections for under-registration and garbage code redistribution algorithms. Levels or estimated time trends might still be affected by systematic problems in selected locations. Forth, ADRD burden might not be estimated accurately due to the lack of detailed disease classification of ADRD of GBD database, such as Alzheimer’s disease, dementia with Lewy bodies, and vascular dementia, our study could not investigate the link between burdens of potential subtypes of ADRD to health inequalities. This will also be the problem as more countries start to experience burden from ADRD. Finally, this framework, however, did not capture true cohort effects, so it’s difficult to discern the underlying factors influencing sex disparities in diseases and how different disease burden indicators changed between the time before and after national government actions.

## Conclusion

This study provides comprehensive updates from prior GBD studies and reveals global, regional, and national health inequalities of ADRD and PD in 204 countries. The global burden of ADRD and PD showed upward trends over the nearly three decades. Burden of ADRD and PD reflected by the health inequality index turned out to be substantially and positively correlated to SDI levels. The health inequality index (SII for AI; CIX for RI) showed a larger difference in 2019 than in 1990. Additionally, we reported disparities in health inequalities across sexes, and identified females had higher disparities in health inequalities than males for ADRD, while showed the opposite for PD. These findings should help to focus prevention and treatment efforts on genders and areas that have experienced inequitable health outcomes.

### Electronic supplementary material

Below is the link to the electronic supplementary material.

Supplementary Material 1

## Data Availability

All data are publicly accessible from the Global Burden of Disease 2019 through the GBD Results tool (http://ghdx.healthdata.org/gbd-results-tool).

## Abbreviations

ADRD: Alzheimer’s disease and related dementias;
AI: absolute index of inequality;
ASRs: age-standardized rates;
ASDRs: age-standardized death rates;
ASIRs: age-standardized incidence rates;
CIs: confidence intervals;
CIX: concentration index;
DALYs: disability-adjusted life years;
EAPCs: estimated annual percentage changes;
GBD: global burden of disease;
GHDx: global health data exchange;
IHME: health metrics and evaluation;
PD: Parkinson’s disease;
RI: relative of inequality index;
SDI: socio-demographic index;
SII: slope index of inequality;
WHO: world health organization;
