# Supplementary material for: Global, regional, and national health inequalities of Alzheimer’s disease and Parkinson’s disease in 204 countries, 1990–2019
Source: Int J Equity Health. 2024 Jun 19;23:125. doi: 10.1186/s12939-024-02212-5 (PMC11188225; doi:10.1186/s12939-024-02212-5)
Supplement: Supplementary file 1 — Supplementary Material 1 [file 12939_2024_2212_MOESM1_ESM.docx]

**eMethods**

**Mortality estimates**

For completely covered regions, vital registration data has been applied to estimate all-cause mortality rates, which are then examined using the demographic equilibrium technique to evaluate subsequent census population counts. These mortality probabilities are converted into age-specific death rates by location, year, and sex applying model life tables. In countries with no other available data, GBD supplements these data with information from police and mortuaries. GBD compiles a sizable database of the cause of death data from vital registration and verbal autopsy investigations, in which relatives are asked a standard set of questions to determine the likely cause of death. Data on injuries and deaths are enhanced. A multitude of categorization systems, whether from specific classifications in some nations or from variants of the International Categorization of Diseases (ICD), give cause of death information. The entirety of the data is matched to a GBD category of disease or injury. The data were assessed by applying the Cause of Death Integration Model (CODEm). This highly systematic tool executes many models on the same data and takes the set of models that best represents all the available input data, after reallocating and correcting for under-registration. All models' statistical performance was evaluated by keeping 30% of the data and assessing how well the models explained the remaining data. The total of all-cause-specific death rates was scaled to all-cause mortality rates for each age, sex, region, and year category to improve the uniformity of the CODEm. We assign a probable cause to every death, therefore all of our estimations of the cause of death are categorical. This indicates that the overall accuracy of all estimations reaches 100% [1]. Indicators designed to be included in the Estimates of Mortality column include Deaths (& Mortality), and years of life lost (YLLs).

**Non-fatal estimates**

The proportions in each sequela were obtained from studies of patient-level datasets, or they were integrated using DisMod-MR 2.1 or meta-analysis [2]. Estimates of years lived with disability (YLDs) that did not yet take comorbidities into account were created by multiplying the prevalence instances of each disease sequela by the relevant disability weights. Using this data, simulations were run to generate hypothetical persons with no, one, or multiple sequelae at the same time for every combination of age, sex, region, and year in order to account for comorbidities. Because providing a combined disability weight value to any person with more than 1, i.e., a year worse than the year lost to death, was avoided, the researchers believed that the disability weights were multiplicative rather than additive. Indicators designed to be included in the Estimates of the non-fatal column include incident cases (& Incidence), prevalent cases (& Prevalence), and years lived with disability (YLDs).

**Case definitions**

For Alzheimer’s disease and related dementias [3], memory loss and other neurological dysfunctions are symptoms of dementia, a progressive, degenerative, and chronic neurological illness. The Diagnostic and Statistical Manual of Mental Disorders III, IV, or V, or the ICD case definitions, are the source of information for GBD 2019. The DSM IV defines many cognitive abnormalities as follows: disturbance in executive functioning, aphasia, apraxia, or agnosia as well as memory impairment; must signify a notable decrease and result in a major impairment in occupational functioning; the course is marked by a continuing and progressive cognitive impairment; cognitive deficiencies may not just occur during delirium; they can also be caused by other mental health issues.

For Parkinson’s disease [3], it is a neurological disorder that is progressive, degenerative, and chronic. It is characterized by tremors, which are the loss of motor control and movement. At least two of the four main symptoms of GBD—tremors/trembling, bradykinesia, rigidity in the limbs and torso, and unstable posture—must be present in order for a case to be diagnosed.

**Risk factors**

To estimate the impact of risk, the collaborators use an alternative counterfactual technique called "What would the burden be if people were exposed to the lowest theoretical level of risk?" Determining the degree of exposure to risk variables that led to the fewest diseases was thus essential. Following an analysis of data on the prevalence of exposure to the risk, the relative risk of all risk-outcome pairings for which there is adequate evidence of causation is determined. 16 Spatiotemporal Gaussian process regression was used in DisMod-MR 2.1 to estimate the prevalence of exposure, or satellite imagery was used in the case of ambient air pollution. Cohort, case-control, and intervention study meta-analyses were used to aggregate relative risk data [4]. Based on the prevalence and relative risk data, population attribution scores concerning the Theoretical Minimum Risk Exposure Level (TMREL) were calculated. Risk must meet certain requirements to be included in the GBD. These include having enough proof of a causal relationship between risk and one or more disease or injury outcomes, proof that effect sizes are more common outside of the populations covered by the epidemiologic study, enough data and methodology to allow estimation of exposure levels by nation and a probability that the risk factor will have an impact on the burden of disease or policy decisions [5].

**Uncertainty intervals (UIs)**

Uncertainty is propagated across all of these calculations by creating 1000 values for each incidence, prevalence, mortality, or DALY estimate and performing aggregation across causes and locations at the level of each of the 1000 values for all intermediate steps in the calculation. The lower and upper bounds of the 95% uncertainty intervals (UI) are the 25^th^ and 975^th^ values of the ordered 1000 values. The significance of a difference is determined if 975 or more of the ordered 1000 differences lie on either side of zero.

**Socio-demographic index (SDI)**

As a measure of social and economic growth, the Socio-demographic Index (SDI) compiles data on the fertility rate, education level, and economics of all nations. This indicator is directly related to health outcomes. To put it straightforwardly, it is the geometric mean of the 0 to 1 index of the mean education among individuals 15 years of age and above (EDU15+), the lag-distributed income (LDI) per capita, and the total fertility rate (TFR) for those under 25 (TFU25) [6]. The SDI value is categorized into five classes, Low SDI, Low-middle SDI, Middle SDI, High-middle SDI, and High SDI, under the boundaries of 0.45, 0.61, 0.69, and 0.81 [7]. Data-accessible websites, https://ghdx.healthdata.org/record/ihme-data/gbd-2019-socio-demographic-index-sdi-1950-2019.

**Reference**

1. **Global, regional, and national age-sex specific mortality for 264 causes of death, 1980-2016: a systematic analysis for the Global Burden of Disease Study 2016.** *Lancet* 2017, **390:**1151-1210.

2. Feigin VL, Nichols E, Alam T, Bannick MS, Beghi E, Blake N, Culpepper WJ, Dorsey ER, Elbaz A, Ellenbogen RG, et al: **Global, regional, and national burden of neurological disorders, 1990–2016: a systematic analysis for the Global Burden of Disease Study 2016.** *The Lancet Neurology* 2019, **18:**459-480.

3. Vos T, Lim SS, Abbafati C, Abbas KM, Abbasi M, Abbasifard M, Abbasi-Kangevari M, Abbastabar H, Abd-Allah F, Abdelalim A, et al: **Global burden of 369 diseases and injuries in 204 countries and territories, 1990–2019: a systematic analysis for the Global Burden of Disease Study 2019.** *The Lancet* 2020, **396:**1204-1222.

4. **Global burden of 87 risk factors in 204 countries and territories, 1990-2019: a systematic analysis for the Global Burden of Disease Study 2019.** *Lancet* 2020, **396:**1223-1249.

5. **Global, regional, and national comparative risk assessment of 84 behavioural, environmental and occupational, and metabolic risks or clusters of risks, 1990-2016: a systematic analysis for the Global Burden of Disease Study 2016.** *Lancet* 2017, **390:**1345-1422.

6. **Global burden of 369 diseases and injuries in 204 countries and territories, 1990-2019: a systematic analysis for the Global Burden of Disease Study 2019.** *Lancet* 2020, **396:**1204-1222.

7. **Global Burden of Disease Collaborative Network. Global Burden of Disease Study 2019 (GBD 2019) Socio-Demographic Index (SDI) 1950–2019** [<https://ghdx.healthdata.org/record/ihme-data/gbd-2019-socio-demographic-index-sdi-1950-2019>]

**eTable 1**. The International Classification of Diseases (ICD) codes mapped to two kinds of neurological disorders in GBD 2019

| The International Classification of Diseases (ICD) codes mapped to two kinds of neurological disorders in GBD 2019 | | | | |
| --- | --- | --- | --- | --- |
| Neurological disorders |  | **ICD-9** |  | **ICD-10** |
| Alzheimer’s disease and related dementias |  | 290-290.9, 294.0-294.9, 331-331.2, 331.6-331.7, 331.82, 331.89-331.9 |  | F00-F02.0, F02.8-F03.91, F06.2, G30-G31.1, G31.8-G32.89 |
| Parkinson's disease |  | 332-332.0 |  | F02.3, G20-G20.9 |

**eTable 2.** The cases of incidence, deaths, and DALYs for Alzheimer’s disease and related dementias in 1990 and 2019, with male-to-female ratios and change percentage.

| Location | Incident cases | | | | | Death cases | | | | | DALYs cases | | | | |
| --- | --- | --- | --- | --- | --- | --- | --- | --- | --- | --- | --- | --- | --- | --- | --- |
|  | 1990 | | 2019 | | Change Percent (%) | 1990 | | 2019 | | Change Percent (%) | 1990 | | 2019 | | Change Percent (%) |
|  | Cases | Male/Female Ratio | Cases | Male/Female Ratio |  | Cases | Male/Female Ratio | Cases | Male/Female Ratio |  | Cases | Male/Female Ratio | Cases | Male/Female Ratio |  |
| Global | 2920985 (2485353 to 3372859) | 0.53 (0.46 to 0.61) | 7236385 (6217239 to 8232672) | 0.59 (0.51 to 0.68) | 147.74 | 560935 (135270 to 1546915) | 0.48 (0.41 to 0.55) | 1623276 (407465 to 4205719) | 0.53 (0.46 to 0.61) | 189.39 | 9663312 (4233955 to 21374779) | 0.54 (0.46 to 0.62) | 25276989 (11204523 to 54558243) | 0.59 (0.52 to 0.67) | 161.58 |
| Sociodemographic index (SDI) |  |  |  |  |  |  |  |  |  |  |  |  |  |  |  |
| High SDI | 1053322 (900538 to 1208885) | 0.44 (0.39 to 0.52) | 2225885 (1933223 to 2505522) | 0.53 (0.46 to 0.62) | 111.32 | 215302 (53618 to 576706) | 0.37 (0.32 to 0.43) | 563216 (148167 to 1394429) | 0.46 (0.4 to 0.53) | 161.59 | 3352394 (1532632 to 7312196) | 0.44 (0.38 to 0.51) | 7655560 (3563493 to 15743216) | 0.52 (0.46 to 0.61) | 128.36 |
| High-middle SDI | 842713 (707776 to 986248) | 0.47 (0.41 to 0.54) | 2013911 (1708382 to 2308751) | 0.54 (0.47 to 0.62) | 138.98 | 151862 (36762 to 415314) | 0.43 (0.38 to 0.5) | 424331 (104344 to 1120445) | 0.47 (0.4 to 0.54) | 179.42 | 2707435 (1192058 to 5916998) | 0.48 (0.42 to 0.56) | 6807505 (3082423 to 14821541) | 0.53 (0.46 to 0.61) | 151.44 |
| Middle SDI | 614379 (524282 to 709550) | 0.63 (0.55 to 0.73) | 1898447 (1618749 to 2181890) | 0.65 (0.57 to 0.75) | 209.00 | 119207 (27985 to 330654) | 0.58 (0.5 to 0.67) | 387516 (93372 to 1026399) | 0.6 (0.52 to 0.69) | 225.08 | 2198619 (915904 to 5125381) | 0.64 (0.55 to 0.73) | 6689285 (2842928 to 15089735) | 0.65 (0.57 to 0.75) | 204.25 |
| Low-middle SDI | 295070 (252580 to 339557) | 0.79 (0.68 to 0.91) | 821293 (704427 to 943578) | 0.73 (0.63 to 0.84) | 178.34 | 53709 (12409 to 151819) | 0.78 (0.68 to 0.89) | 185268 (45622 to 499326) | 0.69 (0.61 to 0.8) | 244.95 | 1011102 (426011 to 2408417) | 0.82 (0.71 to 0.95) | 3072172 (1276081 to 7056377) | 0.73 (0.63 to 0.84) | 203.84 |
| Low SDI | 114030 (97674 to 131229) | 0.79 (0.69 to 0.9) | 273427 (236801 to 312309) | 0.74 (0.64 to 0.84) | 139.79 | 20537 (4677 to 58297) | 0.79 (0.69 to 0.91) | 62096 (15076 to 171251) | 0.71 (0.62 to 0.82) | 202.36 | 388532 (162187 to 912709) | 0.83 (0.72 to 0.96) | 1039872 (429369 to 2448168) | 0.75 (0.66 to 0.86) | 167.64 |
| Region |  |  |  |  |  |  |  |  |  |  |  |  |  |  |  |
| Andean Latin America | 13534 (11583 to 15675) | 0.84 (0.73 to 0.97) | 44044 (37768 to 50206) | 0.79 (0.69 to 0.91) | 225.43 | 3216 (790 to 8733) | 0.86 (0.76 to 0.98) | 11175 (2752 to 28518) | 0.79 (0.68 to 0.91) | 247.48 | 50263 (20825 to 117021) | 0.85 (0.74 to 0.98) | 163614 (69802 to 362966) | 0.81 (0.71 to 0.94) | 225.52 |
| Australasia | 21424 (18272 to 24547) | 0.43 (0.37 to 0.49) | 52430 (44896 to 59599) | 0.53 (0.46 to 0.61) | 144.73 | 4440 (1087 to 11951) | 0.39 (0.34 to 0.45) | 13307 (3458 to 33904) | 0.52 (0.45 to 0.59) | 199.71 | 70313 (31787 to 154557) | 0.45 (0.4 to 0.52) | 183099 (84034 to 390106) | 0.57 (0.5 to 0.65) | 160.41 |
| Caribbean | 17604 (14836 to 20400) | 0.8 (0.69 to 0.93) | 40875 (35109 to 46713) | 0.72 (0.63 to 0.84) | 132.19 | 4094 (1000 to 11345) | 0.82 (0.72 to 0.94) | 10994 (2805 to 27301) | 0.72 (0.62 to 0.83) | 168.54 | 66114 (27549 to 151677) | 0.83 (0.73 to 0.97) | 155718 (65947 to 342864) | 0.75 (0.65 to 0.87) | 135.53 |
| Central Asia | 38810 (32714 to 44638) | 0.46 (0.4 to 0.53) | 50655 (43028 to 58477) | 0.53 (0.46 to 0.61) | 30.52 | 7420 (1825 to 20693) | 0.44 (0.38 to 0.51) | 8947 (2170 to 24591) | 0.47 (0.41 to 0.55) | 20.58 | 121045 (55170 to 265205) | 0.45 (0.4 to 0.52) | 157279 (70959 to 351465) | 0.52 (0.45 to 0.6) | 29.93 |
| Central Europe | 132332 (109495 to 155325) | 0.49 (0.43 to 0.57) | 243264 (203462 to 279725) | 0.49 (0.43 to 0.57) | 83.83 | 23979 (5505 to 67008) | 0.48 (0.42 to 0.55) | 51106 (12307 to 132680) | 0.45 (0.39 to 0.52) | 113.13 | 419000 (188284 to 935936) | 0.5 (0.43 to 0.57) | 794201 (366490 to 1690234) | 0.48 (0.42 to 0.55) | 89.55 |
| Central Latin America | 54573 (46346 to 63261) | 0.81 (0.71 to 0.93) | 182908 (156453 to 209458) | 0.73 (0.63 to 0.83) | 235.16 | 13106 (3195 to 35910) | 0.86 (0.75 to 1) | 51391 (13041 to 130460) | 0.75 (0.66 to 0.86) | 292.12 | 209012 (85186 to 485689) | 0.84 (0.74 to 0.97) | 728751 (299639 to 1628646) | 0.76 (0.66 to 0.88) | 248.66 |
| Central Sub-Saharan Africa | 12463 (10589 to 14513) | 0.62 (0.54 to 0.72) | 32617 (28309 to 37147) | 0.48 (0.41 to 0.55) | 161.71 | 1795 (414 to 5110) | 0.74 (0.64 to 0.85) | 6228 (1544 to 16779) | 0.48 (0.42 to 0.56) | 246.96 | 37878 (16540 to 88422) | 0.72 (0.62 to 0.83) | 112382 (48663 to 258388) | 0.51 (0.44 to 0.59) | 196.69 |
| East Asia | 531620 (451303 to 617552) | 0.57 (0.5 to 0.64) | 1861337 (1570266 to 2153332) | 0.6 (0.53 to 0.69) | 250.13 | 96856 (21813 to 272172) | 0.48 (0.42 to 0.56) | 334678 (79696 to 885891) | 0.5 (0.43 to 0.58) | 245.54 | 1913460 (791138 to 4462799) | 0.58 (0.5 to 0.67) | 6201123 (2778734 to 13575026) | 0.59 (0.51 to 0.68) | 224.08 |
| Eastern Europe | 232393 (189005 to 276680) | 0.31 (0.27 to 0.36) | 359981 (299240 to 417074) | 0.38 (0.33 to 0.43) | 54.90 | 38362 (8752 to 107263) | 0.27 (0.24 to 0.32) | 73082 (17747 to 195332) | 0.33 (0.29 to 0.38) | 90.51 | 677277 (314810 to 1507728) | 0.3 (0.26 to 0.34) | 1141397 (525883 to 2486210) | 0.36 (0.31 to 0.42) | 68.53 |
| Eastern Sub-Saharan Africa | 39896 (34340 to 46019) | 0.68 (0.59 to 0.78) | 92585 (80495 to 105549) | 0.6 (0.53 to 0.7) | 132.07 | 7209 (1651 to 20537) | 0.69 (0.6 to 0.79) | 21430 (5160 to 59015) | 0.58 (0.51 to 0.67) | 197.27 | 135078 (56954 to 313892) | 0.74 (0.64 to 0.83) | 354144 (146347 to 817435) | 0.63 (0.55 to 0.73) | 162.18 |
| High-income Asia Pacific | 168767 (144314 to 196090) | 0.45 (0.39 to 0.51) | 628980 (540697 to 713926) | 0.45 (0.39 to 0.52) | 272.69 | 32094 (7771 to 88167) | 0.46 (0.39 to 0.52) | 184826 (51593 to 444410) | 0.44 (0.39 to 0.5) | 475.89 | 538921 (239608 to 1176431) | 0.5 (0.43 to 0.57) | 2370759 (1102292 to 4847730) | 0.49 (0.42 to 0.55) | 339.91 |
| High-income North America | 417105 (352873 to 478681) | 0.49 (0.43 to 0.57) | 740732 (657407 to 816179) | 0.59 (0.52 to 0.68) | 77.59 | 79541 (20010 to 211672) | 0.39 (0.33 to 0.45) | 160982 (41431 to 396143) | 0.47 (0.41 to 0.54) | 102.39 | 1238021 (592776 to 2625310) | 0.46 (0.41 to 0.53) | 2270345 (1096610 to 4697148) | 0.57 (0.49 to 0.65) | 83.39 |
| North Africa and Middle East | 130152 (110294 to 149526) | 0.86 (0.75 to 0.99) | 361192 (309692 to 413138) | 0.91 (0.79 to 1.05) | 177.52 | 24173 (5791 to 67225) | 0.79 (0.69 to 0.91) | 70483 (17247 to 185789) | 0.88 (0.77 to 1) | 191.58 | 435645 (189151 to 979609) | 0.83 (0.73 to 0.95) | 1208064 (532026 to 2672826) | 0.89 (0.77 to 1.02) | 177.30 |
| Oceania | 1480 (1252 to 1720) | 0.76 (0.66 to 0.88) | 3627 (3089 to 4163) | 0.76 (0.67 to 0.86) | 145.07 | 263 (60 to 717) | 0.75 (0.65 to 0.86) | 673 (157 to 1815) | 0.71 (0.63 to 0.83) | 155.89 | 5316 (2154 to 12582) | 0.8 (0.7 to 0.92) | 12668 (5281 to 29630) | 0.79 (0.68 to 0.91) | 138.30 |
| South Asia | 217938 (186501 to 251282) | 0.97 (0.84 to 1.13) | 678661 (581647 to 781921) | 0.85 (0.73 to 0.98) | 211.40 | 36318 (8199 to 105378) | 1.02 (0.89 to 1.17) | 158114 (37653 to 437057) | 0.8 (0.69 to 0.92) | 335.36 | 725766 (300323 to 1729469) | 1.01 (0.88 to 1.17) | 2617558 (1061033 to 6225230) | 0.83 (0.72 to 0.96) | 260.66 |
| Southeast Asia | 151982 (130855 to 173933) | 0.59 (0.51 to 0.68) | 402323 (347100 to 456730) | 0.55 (0.48 to 0.64) | 164.72 | 32345 (7671 to 89723) | 0.52 (0.45 to 0.6) | 93396 (22132 to 241959) | 0.51 (0.44 to 0.58) | 188.75 | 573670 (234420 to 1323275) | 0.58 (0.51 to 0.66) | 1551369 (634693 to 3426746) | 0.55 (0.48 to 0.64) | 170.43 |
| Southern Latin America | 37271 (31205 to 43622) | 0.55 (0.48 to 0.63) | 82527 (70038 to 94751) | 0.53 (0.47 to 0.61) | 121.42 | 7046 (1674 to 19566) | 0.5 (0.43 to 0.58) | 18346 (4478 to 48830) | 0.47 (0.41 to 0.54) | 160.37 | 116329 (52710 to 256958) | 0.56 (0.48 to 0.63) | 271093 (123669 to 586534) | 0.53 (0.46 to 0.62) | 133.04 |
| Southern Sub-Saharan Africa | 19044 (16414 to 21780) | 0.48 (0.42 to 0.55) | 38365 (33144 to 44024) | 0.44 (0.38 to 0.51) | 101.45 | 3909 (926 to 10657) | 0.46 (0.4 to 0.53) | 7979 (1874 to 22083) | 0.41 (0.36 to 0.47) | 104.12 | 63247 (27074 to 145852) | 0.49 (0.43 to 0.56) | 130478 (56316 to 298140) | 0.45 (0.39 to 0.52) | 106.30 |
| Tropical Latin America | 69561 (59574 to 79711) | 0.64 (0.56 to 0.75) | 236870 (206086 to 268576) | 0.66 (0.57 to 0.77) | 240.52 | 13743 (3270 to 37800) | 0.66 (0.57 to 0.76) | 55855 (14157 to 144037) | 0.62 (0.54 to 0.72) | 306.43 | 242086 (104317 to 551880) | 0.68 (0.6 to 0.78) | 873878 (384944 to 1910951) | 0.66 (0.57 to 0.76) | 260.98 |
| Western Europe | 568773 (487919 to 651556) | 0.4 (0.35 to 0.46) | 1009029 (857524 to 1155105) | 0.5 (0.44 to 0.58) | 77.40 | 120865 (29291 to 320938) | 0.35 (0.31 to 0.41) | 264766 (67081 to 676335) | 0.45 (0.39 to 0.52) | 119.06 | 1854194 (840012 to 4046219) | 0.41 (0.35 to 0.47) | 3582944 (1636179 to 7579612) | 0.51 (0.44 to 0.58) | 93.23 |
| Western Sub-Saharan Africa | 44264 (37956 to 50819) | 0.72 (0.62 to 0.82) | 93382 (80981 to 106420) | 0.72 (0.62 to 0.83) | 110.97 | 10161 (2404 to 29552) | 0.62 (0.54 to 0.72) | 25519 (6094 to 68214) | 0.72 (0.63 to 0.83) | 151.15 | 170677 (68670 to 419728) | 0.7 (0.61 to 0.81) | 396124 (155275 to 929088) | 0.75 (0.66 to 0.86) | 132.09 |

**Abbreviations**: DALYs, disability-adjusted life-years;

Below the Cases and Male/Female Ratio column are their estimated values and 95% uncertainty intervals; Change Percent represents the percentage change in absolute cases from 1990 to 2019.

**eTable 3.** The cases of incidence, deaths, and DALYs for Parkinson’s disease in 1990 and 2019, with male-to-female ratios and change percentage.

| Location | Incident cases | | | | | Death cases | | | | | DALYs cases | | | | |
| --- | --- | --- | --- | --- | --- | --- | --- | --- | --- | --- | --- | --- | --- | --- | --- |
|  | 1990 | | 2019 | | Change Percent (%) | 1990 | | 2019 | | Change Percent (%) | 1990 | | 2019 | | Change Percent (%) |
|  | Cases | Male/Female Ratio | Cases | Male/Female Ratio |  | Cases | Male/Female Ratio | Cases | Male/Female Ratio |  | Cases | Male/Female Ratio | Cases | Male/Female Ratio |  |
| Global | 416474 (362669 to 473210) | 1.16 (1 to 1.33) | 1081723 (953265 to 1211202) | 1.48 (1.28 to 1.7) | 159.73 | 147238 (137335 to 158063) | 1.16 (1 to 1.33) | 362907 (326855 to 388200) | 1.35 (1.18 to 1.56) | 146.48 | 2749554 (2541530 to 2992889) | 1.2 (1.03 to 1.38) | 6292616 (5769210 to 6827207) | 1.4 (1.22 to 1.61) | 128.86 |
| Sociodemographic index (SDI) |  |  |  |  |  |  |  |  |  |  |  |  |  |  |  |
| High SDI | 121168 (107649 to 135904) | 1.13 (0.99 to 1.32) | 330609 (297949 to 362338) | 2 (1.75 to 2.33) | 172.85 | 43799 (40355 to 45436) | 1.11 (0.96 to 1.29) | 104440 (91890 to 111339) | 1.48 (1.29 to 1.69) | 138.45 | 746284 (693520 to 798411) | 1.17 (1.01 to 1.34) | 1604208 (1454060 to 1725513) | 1.52 (1.32 to 1.76) | 114.96 |
| High-middle SDI | 125883 (108929 to 143926) | 1.04 (0.91 to 1.2) | 272315 (236518 to 309170) | 1.29 (1.13 to 1.49) | 116.32 | 42606 (39920 to 44831) | 1.04 (0.91 to 1.19) | 88876 (80363 to 95439) | 1.25 (1.08 to 1.44) | 108.6 | 794918 (735975 to 856237) | 1.06 (0.92 to 1.23) | 1553400 (1403033 to 1701395) | 1.31 (1.14 to 1.51) | 95.42 |
| Middle SDI | 102759 (88180 to 117739) | 1.23 (1.06 to 1.41) | 302119 (258634 to 345464) | 1.34 (1.16 to 1.53) | 194.01 | 34061 (31381 to 37188) | 1.15 (1 to 1.33) | 95947 (86169 to 105224) | 1.34 (1.16 to 1.54) | 181.69 | 682469 (621660 to 756663) | 1.21 (1.05 to 1.39) | 1800862 (1629327 to 1993459) | 1.39 (1.21 to 1.59) | 163.87 |
| Low-middle SDI | 49501 (42933 to 56174) | 1.33 (1.16 to 1.53) | 135131 (117778 to 153296) | 1.29 (1.13 to 1.48) | 172.99 | 19687 (17039 to 23672) | 1.44 (1.26 to 1.64) | 56172 (50170 to 62978) | 1.31 (1.15 to 1.51) | 185.33 | 385776 (335031 to 459989) | 1.44 (1.26 to 1.68) | 1012326 (908057 to 1129431) | 1.33 (1.16 to 1.54) | 162.41 |
| Low SDI | 16943 (14852 to 19179) | 1.4 (1.21 to 1.61) | 41045 (36257 to 46044) | 1.29 (1.12 to 1.49) | 142.25 | 7003 (5752 to 9354) | 1.59 (1.37 to 1.83) | 17273 (15146 to 20152) | 1.36 (1.18 to 1.56) | 146.65 | 138637 (116485 to 179425) | 1.59 (1.38 to 1.83) | 318441 (282136 to 364301) | 1.38 (1.21 to 1.59) | 129.69 |
| Region |  |  |  |  |  |  |  |  |  |  |  |  |  |  |  |
| Andean Latin America | 1950 (1751 to 2138) | 1.4 (1.22 to 1.59) | 6841 (6242 to 7439) | 1.45 (1.26 to 1.65) | 250.82 | 825 (732 to 964) | 1.46 (1.27 to 1.65) | 2772 (2275 to 3286) | 1.41 (1.23 to 1.62) | 236 | 14412 (12845 to 16560) | 1.53 (1.33 to 1.76) | 44791 (37546 to 52145) | 1.47 (1.28 to 1.66) | 210.79 |
| Australasia | 3257 (2951 to 3622) | 1.41 (1.23 to 1.62) | 7693 (6778 to 8751) | 1.79 (1.57 to 2.06) | 136.2 | 1040 (961 to 1087) | 1.42 (1.24 to 1.63) | 2806 (2430 to 3022) | 1.86 (1.62 to 2.12) | 169.81 | 19086 (17578 to 20605) | 1.47 (1.29 to 1.7) | 45277 (40461 to 49754) | 1.88 (1.64 to 2.16) | 137.23 |
| Caribbean | 2455 (2243 to 2689) | 1.51 (1.32 to 1.74) | 5936 (5409 to 6480) | 1.46 (1.28 to 1.68) | 141.79 | 990 (914 to 1052) | 1.48 (1.3 to 1.71) | 2466 (2143 to 2782) | 1.37 (1.18 to 1.57) | 149.09 | 17231 (15971 to 18688) | 1.52 (1.31 to 1.76) | 40657 (35860 to 45818) | 1.46 (1.28 to 1.69) | 135.95 |
| Central Asia | 4218 (3784 to 4688) | 0.95 (0.83 to 1.09) | 7083 (6330 to 7878) | 1.08 (0.94 to 1.24) | 67.92 | 1439 (1274 to 1758) | 0.89 (0.77 to 1.03) | 2588 (2353 to 2822) | 0.97 (0.85 to 1.12) | 79.85 | 25879 (23094 to 30139) | 0.92 (0.8 to 1.05) | 46623 (42387 to 51144) | 1.06 (0.91 to 1.22) | 80.16 |
| Central Europe | 16598 (15046 to 18270) | 0.99 (0.86 to 1.15) | 26312 (24211 to 28472) | 1.09 (0.95 to 1.27) | 58.53 | 6402 (6038 to 6595) | 1.01 (0.88 to 1.16) | 11152 (9802 to 12371) | 1.05 (0.9 to 1.21) | 74.2 | 112034 (105568 to 118927) | 1.04 (0.89 to 1.19) | 179385 (159197 to 198302) | 1.11 (0.97 to 1.26) | 60.12 |
| Central Latin America | 7455 (6650 to 8257) | 1.41 (1.22 to 1.64) | 25324 (22670 to 27959) | 1.39 (1.2 to 1.61) | 239.69 | 2772 (2533 to 2889) | 1.36 (1.19 to 1.59) | 9790 (8494 to 11147) | 1.35 (1.18 to 1.55) | 253.17 | 48668 (45404 to 51861) | 1.41 (1.23 to 1.62) | 163356 (144121 to 184002) | 1.41 (1.23 to 1.63) | 235.65 |
| Central Sub-Saharan Africa | 1411 (1221 to 1609) | 1.37 (1.2 to 1.59) | 3477 (3018 to 3967) | 1.14 (1 to 1.32) | 146.42 | 561 (460 to 730) | 1.72 (1.51 to 1.99) | 1325 (1079 to 1696) | 1.15 (1 to 1.33) | 136.19 | 12130 (10032 to 15535) | 1.64 (1.44 to 1.91) | 26743 (21990 to 33355) | 1.22 (1.05 to 1.4) | 120.47 |
| East Asia | 103188 (86356 to 121059) | 1.22 (1.07 to 1.42) | 311857 (260767 to 362953) | 1.42 (1.24 to 1.64) | 202.22 | 33015 (29577 to 37001) | 1.13 (0.99 to 1.31) | 80336 (68611 to 91303) | 1.52 (1.33 to 1.78) | 143.33 | 689675 (613978 to 771124) | 1.19 (1.04 to 1.38) | 1616842 (1392717 to 1855684) | 1.52 (1.32 to 1.75) | 134.44 |
| Eastern Europe | 28412 (23773 to 33451) | 0.7 (0.61 to 0.8) | 36227 (30437 to 42266) | 0.95 (0.83 to 1.09) | 27.51 | 9405 (8711 to 9915) | 0.61 (0.53 to 0.7) | 14364 (12813 to 15709) | 0.74 (0.63 to 0.85) | 52.73 | 173151 (157990 to 188320) | 0.65 (0.56 to 0.75) | 241196 (216886 to 266648) | 0.83 (0.72 to 0.96) | 39.3 |
| Eastern Sub-Saharan Africa | 4840 (4251 to 5492) | 1.52 (1.32 to 1.73) | 10664 (9417 to 12058) | 1.36 (1.19 to 1.55) | 120.33 | 2031 (1636 to 2830) | 1.64 (1.42 to 1.88) | 4608 (3931 to 6741) | 1.37 (1.19 to 1.59) | 126.88 | 41197 (33614 to 55783) | 1.67 (1.46 to 1.89) | 86283 (74469 to 116552) | 1.45 (1.25 to 1.67) | 109.44 |
| High-income Asia Pacific | 14848 (12576 to 17259) | 1.09 (0.94 to 1.25) | 44118 (37490 to 50796) | 1.2 (1.04 to 1.38) | 197.13 | 5744 (5271 to 6289) | 1.15 (1 to 1.32) | 20730 (17091 to 22774) | 1.19 (1.03 to 1.37) | 260.9 | 100736 (93272 to 109457) | 1.16 (1.01 to 1.34) | 305661 (264553 to 333623) | 1.23 (1.07 to 1.4) | 203.43 |
| High-income North America | 41413 (34727 to 48371) | 1.18 (1.03 to 1.37) | 164043 (142421 to 186992) | 3.13 (2.73 to 3.61) | 296.11 | 14442 (13082 to 15077) | 1.07 (0.92 to 1.23) | 36338 (32285 to 38461) | 1.63 (1.42 to 1.88) | 151.61 | 242374 (223305 to 260529) | 1.16 (1.02 to 1.34) | 556994 (511705 to 590395) | 1.7 (1.48 to 1.97) | 129.81 |
| North Africa and Middle East | 14634 (12987 to 16358) | 1.42 (1.24 to 1.64) | 42804 (38332 to 47338) | 1.47 (1.28 to 1.69) | 192.5 | 6006 (5245 to 7808) | 1.44 (1.25 to 1.67) | 16784 (14607 to 21649) | 1.5 (1.3 to 1.7) | 179.45 | 113988 (99982 to 140731) | 1.41 (1.23 to 1.61) | 300698 (266277 to 365360) | 1.46 (1.27 to 1.68) | 163.8 |
| Oceania | 394 (344 to 449) | 1.71 (1.49 to 1.97) | 869 (763 to 977) | 1.64 (1.43 to 1.89) | 120.56 | 150 (98 to 187) | 1.79 (1.56 to 2.07) | 338 (229 to 411) | 1.6 (1.41 to 1.86) | 125.33 | 3157 (2118 to 3922) | 1.8 (1.57 to 2.08) | 6929 (4603 to 8457) | 1.64 (1.41 to 1.9) | 119.48 |
| South Asia | 42260 (35824 to 49078) | 1.47 (1.28 to 1.69) | 128494 (108403 to 149539) | 1.33 (1.16 to 1.53) | 204.06 | 17807 (14555 to 22546) | 1.64 (1.42 to 1.86) | 54968 (47446 to 63571) | 1.34 (1.17 to 1.55) | 208.69 | 348120 (289627 to 434162) | 1.6 (1.4 to 1.86) | 984634 (854639 to 1124852) | 1.36 (1.19 to 1.59) | 182.84 |
| Southeast Asia | 24644 (21765 to 27809) | 1.09 (0.94 to 1.26) | 65111 (57547 to 73056) | 1.1 (0.95 to 1.27) | 164.21 | 7793 (6680 to 8652) | 1.02 (0.89 to 1.19) | 22724 (19234 to 25733) | 1.07 (0.92 to 1.22) | 191.6 | 155093 (133287 to 173801) | 1.1 (0.95 to 1.25) | 421113 (363277 to 473855) | 1.17 (1.02 to 1.35) | 171.52 |
| Southern Latin America | 5489 (4995 to 6106) | 1.51 (1.33 to 1.75) | 10428 (9418 to 11482) | 1.64 (1.44 to 1.9) | 89.98 | 2135 (1997 to 2240) | 1.49 (1.29 to 1.71) | 4726 (4275 to 5018) | 1.48 (1.29 to 1.7) | 121.36 | 36188 (33918 to 38608) | 1.54 (1.34 to 1.75) | 72474 (66659 to 77506) | 1.6 (1.4 to 1.87) | 100.27 |
| Southern Sub-Saharan Africa | 1924 (1667 to 2191) | 1.14 (1 to 1.31) | 4528 (3882 to 5218) | 1.03 (0.9 to 1.18) | 135.34 | 638 (572 to 711) | 1 (0.87 to 1.15) | 1680 (1526 to 1806) | 0.9 (0.79 to 1.03) | 163.32 | 11341 (10260 to 12642) | 1.16 (1.02 to 1.33) | 28753 (26239 to 31169) | 1.06 (0.93 to 1.22) | 153.53 |
| Tropical Latin America | 7878 (6607 to 9180) | 1.24 (1.08 to 1.4) | 25466 (21496 to 29590) | 1.13 (0.99 to 1.28) | 223.25 | 2958 (2721 to 3096) | 1.2 (1.04 to 1.38) | 9778 (8642 to 10491) | 1.08 (0.94 to 1.26) | 230.56 | 53427 (49817 to 57150) | 1.3 (1.14 to 1.5) | 163658 (148827 to 177156) | 1.2 (1.04 to 1.39) | 206.32 |
| Western Europe | 82229 (74555 to 90462) | 1.06 (0.93 to 1.23) | 138245 (123835 to 150578) | 1.35 (1.18 to 1.57) | 68.12 | 28148 (26186 to 29112) | 1.17 (1.02 to 1.35) | 55507 (49083 to 59073) | 1.5 (1.3 to 1.72) | 97.2 | 480520 (445363 to 516003) | 1.16 (1.01 to 1.34) | 841646 (761497 to 910966) | 1.49 (1.3 to 1.71) | 75.15 |
| Western Sub-Saharan Africa | 6976 (6154 to 7828) | 1.17 (1.01 to 1.35) | 16202 (14535 to 17991) | 1.16 (1 to 1.33) | 132.25 | 2935 (2526 to 3477) | 1.01 (0.88 to 1.16) | 7125 (6120 to 7997) | 1.13 (0.99 to 1.28) | 142.76 | 51148 (44609 to 59640) | 1.14 (1 to 1.32) | 118905 (103655 to 134224) | 1.19 (1.05 to 1.38) | 132.47 |

**Abbreviations**: DALYs, disability-adjusted life-years;

Below the Cases and Male/Female Ratio column are their estimated values and 95% uncertainty intervals; Change Percent represents the percentage change in absolute cases from 1990 to 2019.

**eTable 4.** The age-standardized rates per 100 000 people of incidence, deaths, and DALYs for Alzheimer’s disease and related dementias in 1990 and 2019, with male-to-female ratios and estimated annual percentage changes.

| Location | Age-standardized incidence rates | | | | | Age-standardized death rates | | | | | Age-standardized DALYs rates | | | | |
| --- | --- | --- | --- | --- | --- | --- | --- | --- | --- | --- | --- | --- | --- | --- | --- |
|  | 1990 | | 2019 | | EAPC (%) | 1990 | | 2019 | | EAPC (%) | 1990 | | 2019 | | EAPC (%) |
|  | Rates | Male/Female Ratio | Rates | Male/Female Ratio |  | Rates | Male/Female Ratio | Rates | Male/Female Ratio |  | Rates | Male/Female Ratio | Rates | Male/Female Ratio |  |
| Global | 93.58 (80.12 to 106.7) | 0.8 (0.69 to 0.92) | 94.99 (81.59 to 107.86) | 0.81 (0.7 to 0.93) | 0.31 (0.11 to 0.5) | 22.24 (5.5 to 59.98) | 0.84 (0.73 to 0.96) | 22.92 (5.83 to 59.2) | 0.86 (0.75 to 0.99) | 0.21 (0.14 to 0.28) | 326.71 (143.33 to 731.03) | 0.83 (0.72 to 0.96) | 338.64 (151.02 to 731.27) | 0.85 (0.74 to 0.96) | 0.43 (0.27 to 0.59) |
| Sociodemographic index (SDI) |  |  |  |  |  |  |  |  |  |  |  |  |  |  |  |
| High SDI | 99.64 (86.19 to 113.09) | 0.8 (0.69 to 0.92) | 100.57 (87.24 to 113.37) | 0.78 (0.68 to 0.9) | 0.33 (0.16 to 0.51) | 21.96 (5.49 to 57.5) | 0.82 (0.7 to 0.95) | 22.66 (5.89 to 56.63) | 0.84 (0.73 to 0.96) | 0.36 (0.25 to 0.46) | 324.07 (147.34 to 705.64) | 0.83 (0.71 to 0.96) | 332.4 (155 to 688.29) | 0.83 (0.73 to 0.97) | 0.53 (0.35 to 0.7) |
| High-middle SDI | 97.55 (82.44 to 111.71) | 0.82 (0.72 to 0.96) | 101.68 (86.58 to 116.12) | 0.83 (0.72 to 0.97) | 0.41 (0.19 to 0.63) | 22.53 (5.54 to 60.52) | 0.88 (0.77 to 1.01) | 22.8 (5.68 to 60.14) | 0.87 (0.76 to 1.01) | 0.13 (0.09 to 0.18) | 335.16 (148.39 to 736.45) | 0.85 (0.74 to 0.99) | 348.46 (157.71 to 754.37) | 0.86 (0.75 to 0.99) | 0.1 (0.07 to 0.14) |
| Middle SDI | 88.38 (74.76 to 101.4) | 0.81 (0.7 to 0.93) | 93.29 (79.52 to 106.76) | 0.83 (0.72 to 0.95) | 0.41 (0.18 to 0.65) | 23.02 (5.52 to 63.87) | 0.83 (0.72 to 0.96) | 23.21 (5.69 to 60.43) | 0.86 (0.75 to 0.99) | 0.22 (0.11 to 0.33) | 335.33 (139.18 to 770.43) | 0.82 (0.71 to 0.94) | 346.11 (148.62 to 770.18) | 0.84 (0.73 to 0.96) | 0.28 (0.21 to 0.35) |
| Low-middle SDI | 77.31 (65.89 to 88.63) | 0.85 (0.73 to 0.98) | 77.07 (65.7 to 88.14) | 0.88 (0.76 to 1.01) | 0.18 (0.01 to 0.34) | 20.12 (4.77 to 55.72) | 0.88 (0.77 to 1) | 21.61 (5.22 to 56.36) | 0.91 (0.79 to 1.04) | 0.82 (0.64 to 1) | 287.22 (118.6 to 672.6) | 0.88 (0.76 to 1.02) | 304.76 (126.52 to 702.3) | 0.89 (0.77 to 1.03) | 0.45 (0.18 to 0.72) |
| Low SDI | 78.7 (67.02 to 90.5) | 0.84 (0.73 to 0.97) | 76.97 (66.01 to 87.9) | 0.85 (0.74 to 0.98) | 0.12 (-0.03 to 0.27) | 20.9 (4.93 to 58.1) | 0.89 (0.77 to 1.02) | 23.03 (5.64 to 62.49) | 0.88 (0.76 to 1.01) | 0.72 (0.49 to 0.95) | 295.19 (121.35 to 694.71) | 0.87 (0.75 to 1) | 315.76 (129.02 to 742.9) | 0.86 (0.75 to 0.99) | 0.39 (0.24 to 0.54) |
| Region |  |  |  |  |  |  |  |  |  |  |  |  |  |  |  |
| Andean Latin America | 80.97  (68.97 to 93.44) | 0.92 (0.8 to 1.05) | 83.48 (71.56 to 95.52) | 0.91 (0.8 to 1.05) | 0.06 (0.02 to 0.1) | 21.48 (5.26 to 58.33) | 0.93 (0.81 to 1.06) | 21.76 (5.35 to 55.53) | 0.96 (0.83 to 1.11) | 0.4 (0.19 to 0.61) | 306.69 (127.33 to 718.29) | 0.93 (0.81 to 1.07) | 312.67 (133.67 to 691.68) | 0.95 (0.83 to 1.09) | 0.31 (0.14 to 0.48) |
| Australasia | 96.01 (82.52 to 109.28) | 0.7 (0.61 to 0.82) | 93.84 (80.46 to 106.76) | 0.71 (0.62 to 0.82) | 0.08 (-0.03 to 0.18) | 22.57 (5.6 to 59.73) | 0.79 (0.69 to 0.91) | 22.08 (5.71 to 56.53) | 0.8 (0.7 to 0.91) | 0.07 (0.02 to 0.11) | 326.93 (147.01 to 726.32) | 0.78 (0.68 to 0.9) | 319.88 (146.31 to 681.98) | 0.79 (0.69 to 0.91) | 0.31 (0 to 0.61) |
| Caribbean | 77.69 (66.39 to 89.43) | 0.93 (0.81 to 1.08) | 78.42 (67.22 to 89.79) | 0.92 (0.8 to 1.07) | 0.24 (0.07 to 0.42) | 20.59 (5.04 to 55.72) | 0.99 (0.87 to 1.14) | 20.75 (5.26 to 51.95) | 1 (0.86 to 1.15) | -0.23 (-0.32 to -0.13) | 295.98 (122.92 to 684.98) | 0.97 (0.85 to 1.13) | 299.45 (126.93 to 661.71) | 0.97 (0.84 to 1.12) | 0.37 (0.17 to 0.57) |
| Central Asia | 101.63 (86 to 116.8) | 0.91 (0.78 to 1.05) | 102.74 (87.44 to 117.42) | 0.9 (0.78 to 1.04) | 0.13 (0.06 to 0.2) | 21.61 (5.35 to 59.48) | 0.96 (0.83 to 1.11) | 23.45 (5.72 to 64.46) | 0.97 (0.85 to 1.13) | 0.18 (0.11 to 0.25) | 326.32 (149.38 to 721.24) | 0.93 (0.81 to 1.07) | 345.47 (155.28 to 775.08) | 0.94 (0.81 to 1.08) | 0.42 (0.2 to 0.63) |
| Central Europe | 104.04 (87.55 to 119.69) | 0.85 (0.75 to 0.98) | 106.31 (89.8 to 121.85) | 0.85 (0.75 to 0.99) | 0.15 (0.1 to 0.2) | 23.43 (5.7 to 64.73) | 0.93 (0.81 to 1.07) | 23.02 (5.54 to 59.38) | 0.93 (0.8 to 1.08) | -0.35 (-0.63 to -0.06) | 349.87 (156.52 to 778.96) | 0.88 (0.76 to 1.01) | 348.75 (160.87 to 734.47) | 0.88 (0.76 to 1) | 0.83 (0.45 to 1.2) |
| Central Latin America | 83.83 (71.35 to 96.9) | 0.91 (0.79 to 1.05) | 82.9 (70.84 to 95.18) | 0.91 (0.79 to 1.04) | 0.29 (0.06 to 0.51) | 23.44 (5.76 to 63.72) | 0.96 (0.84 to 1.12) | 23.84 (6.04 to 60.61) | 0.98 (0.86 to 1.13) | 0.24 (0.12 to 0.36) | 329.17 (133.63 to 765.75) | 0.94 (0.82 to 1.08) | 333.23 (136.24 to 747.25) | 0.96 (0.84 to 1.11) | -0.07 (-0.19 to 0.06) |
| Central Sub-Saharan Africa | 96.41 (83.03 to 110.18) | 0.76 (0.66 to 0.86) | 98.35 (85.22 to 112.11) | 0.74 (0.64 to 0.84) | 0.36 (0.14 to 0.57) | 23.66 (5.51 to 67.36) | 0.91 (0.79 to 1.05) | 26.57 (6.67 to 71.45) | 0.89 (0.77 to 1.03) | 0.08 (-0.2 to 0.36) | 347.02 (149.92 to 796.7) | 0.84 (0.73 to 0.97) | 380.57 (165.07 to 857.23) | 0.82 (0.71 to 0.94) | 0.34 (0.15 to 0.53) |
| East Asia | 90.36 (76.22 to 104.02) | 0.75 (0.66 to 0.87) | 103.32 (87.6 to 118.29) | 0.78 (0.68 to 0.9) | 0.71 (0.41 to 1) | 23.3 (5.39 to 63.68) | 0.75 (0.65 to 0.86) | 23.19 (5.63 to 61.19) | 0.81 (0.71 to 0.94) | 0.02 (-0.03 to 0.07) | 347.44 (143.95 to 798.77) | 0.76 (0.66 to 0.88) | 366.61 (164.75 to 789.1) | 0.8 (0.69 to 0.92) | 0.08 (-0.12 to 0.29) |
| Eastern Europe | 98.28 (81.88 to 113.72) | 0.9 (0.78 to 1.04) | 101.93 (85.52 to 116.99) | 0.89 (0.78 to 1.03) | 0.31 (0.19 to 0.43) | 20.29 (4.82 to 55.98) | 0.96 (0.84 to 1.11) | 21.55 (5.25 to 57.33) | 0.95 (0.83 to 1.09) | 0.07 (-0.08 to 0.23) | 305.52 (140.31 to 679.46) | 0.92 (0.79 to 1.05) | 324.8 (149.89 to 705.23) | 0.91 (0.79 to 1.05) | 0.44 (0.21 to 0.67) |
| Eastern Sub-Saharan Africa | 85.81 (73.42 to 98.23) | 0.77 (0.67 to 0.88) | 85.08 (73.42 to 96.76) | 0.77 (0.67 to 0.89) | 0.2 (0.03 to 0.38) | 22.95 (5.4 to 62.79) | 0.83 (0.72 to 0.96) | 26.25 (6.52 to 69.47) | 0.83 (0.72 to 0.96) | -0.05 (-0.08 to -0.03) | 323.31 (135.15 to 746.55) | 0.81 (0.71 to 0.91) | 356 (144.97 to 829.93) | 0.8 (0.71 to 0.93) | 0.66 (0.34 to 0.98) |
| High-income Asia Pacific | 96.86 (83.48 to 110.97) | 0.74 (0.64 to 0.84) | 108.9 (93.99 to 123.75) | 0.72 (0.62 to 0.82) | 0.58 (0.5 to 0.67) | 21.86 (5.4 to 58.45) | 0.85 (0.73 to 0.97) | 27 (7.46 to 65.44) | 0.89 (0.78 to 1.01) | 0.15 (-0.13 to 0.43) | 322.6 (143.99 to 710.51) | 0.82 (0.72 to 0.95) | 385.38 (179.3 to 791.75) | 0.84 (0.73 to 0.96) | 0.29 (-0.11 to 0.68) |
| High-income North America | 111.1 (94.6 to 126.89) | 0.86 (0.75 to 1) | 105.77 (93.77 to 116.93) | 0.85 (0.74 to 0.98) | 0.2 (0.01 to 0.39) | 21.38 (5.41 to 56.38) | 0.83 (0.71 to 0.95) | 20.87 (5.34 to 52.13) | 0.81 (0.71 to 0.94) | 0.16 (0.11 to 0.21) | 330.78 (158 to 700.92) | 0.87 (0.76 to 1) | 317.66 (154.02 to 653.15) | 0.86 (0.75 to 1) | 0.07 (-0.01 to 0.16) |
| North Africa and Middle East | 109.25 (92.75 to 125.03) | 0.93 (0.81 to 1.08) | 110.17 (93.94 to 125.62) | 0.92 (0.8 to 1.06) | 0.25 (0.09 to 0.41) | 26.12 (6.35 to 72.64) | 0.92 (0.8 to 1.07) | 25.52 (6.34 to 67.06) | 0.9 (0.78 to 1.02) | -0.01 (-0.07 to 0.06) | 391.32 (170.34 to 883.07) | 0.92 (0.8 to 1.05) | 386.96 (171.95 to 848.51) | 0.89 (0.78 to 1.03) | 0.04 (0.02 to 0.06) |
| Oceania | 88.33 (74.28 to 101.85) | 0.8 (0.7 to 0.9) | 86.26 (73.29 to 98.98) | 0.8 (0.7 to 0.91) | 0.21 (-0.02 to 0.45) | 22.17 (5.12 to 60.7) | 0.89 (0.78 to 1.03) | 21.52 (5.08 to 58.96) | 0.86 (0.75 to 0.99) | -0.14 (-0.17 to -0.12) | 332.94 (136.7 to 765.18) | 0.85 (0.74 to 0.98) | 318.77 (133.77 to 726.91) | 0.84 (0.72 to 0.97) | 0.2 (0.02 to 0.37) |
| South Asia | 64.17 (54.61 to 73.81) | 0.93 (0.8 to 1.07) | 63.6 (54.2 to 72.95) | 0.94 (0.81 to 1.09) | 0.22 (0.01 to 0.42) | 16.79 (3.83 to 48.06) | 0.95 (0.83 to 1.08) | 19.17 (4.62 to 52.06) | 0.93 (0.81 to 1.07) | 0.34 (0.28 to 0.39) | 238.2 (96.13 to 575.6) | 0.96 (0.83 to 1.11) | 262.09 (105.55 to 617.38) | 0.94 (0.82 to 1.09) | 0.49 (0.25 to 0.74) |
| Southeast Asia | 84.62 (72.1 to 97.18) | 0.77 (0.67 to 0.88) | 84.8 (72.56 to 96.84) | 0.76 (0.66 to 0.87) | 0.32 (0.09 to 0.56) | 22.83 (5.48 to 62.35) | 0.75 (0.65 to 0.87) | 23.62 (5.72 to 60.72) | 0.77 (0.67 to 0.89) | -0.24 (-0.47 to -0.01) | 333.58 (136.46 to 764.32) | 0.75 (0.66 to 0.86) | 342.18 (141.51 to 765.11) | 0.77 (0.67 to 0.89) | 0.92 (0.79 to 1.05) |
| Southern Latin America | 92.71 (78.34 to 106.93) | 0.82 (0.71 to 0.94) | 95.18 (80.96 to 109.17) | 0.83 (0.72 to 0.95) | 0.24 (0.13 to 0.35) | 20.86 (5.05 to 56.22) | 0.85 (0.73 to 0.97) | 21.03 (5.13 to 56.08) | 0.85 (0.75 to 0.98) | -0.03 (-0.08 to 0.02) | 304.44 (137 to 674.68) | 0.86 (0.75 to 0.98) | 311.86 (142.12 to 674.17) | 0.87 (0.76 to 1.02) | 0.48 (0.32 to 0.64) |
| Southern Sub-Saharan Africa | 90.15 (77.17 to 103.33) | 0.79 (0.68 to 0.9) | 89.95 (77.28 to 102.68) | 0.8 (0.69 to 0.92) | 0.21 (0.04 to 0.38) | 21.95 (5.17 to 59.26) | 0.87 (0.76 to 1) | 22.87 (5.49 to 62.53) | 0.88 (0.77 to 1.01) | 0.56 (0.52 to 0.59) | 313.71 (135.09 to 712.18) | 0.82 (0.72 to 0.95) | 323.81 (138.29 to 740.97) | 0.83 (0.73 to 0.97) | 0.67 (0.46 to 0.88) |
| Tropical Latin America | 102.3 (88.03 to 116.99) | 0.82 (0.72 to 0.95) | 104.09 (90.49 to 118.13) | 0.91 (0.8 to 1.06) | 0.29 (0.13 to 0.45) | 26.64 (6.57 to 71.11) | 0.92 (0.8 to 1.06) | 25.57 (6.51 to 66.21) | 0.97 (0.85 to 1.13) | 0.04 (-0.12 to 0.21) | 384.41 (163.84 to 871.32) | 0.89 (0.78 to 1.02) | 390.88 (172.01 to 855.99) | 0.95 (0.82 to 1.1) | 0.28 (0.2 to 0.35) |
| Western Europe | 93.49 (81.02 to 105.5) | 0.75 (0.65 to 0.86) | 91.52 (78.13 to 104.43) | 0.75 (0.66 to 0.87) | 0.24 (0.01 to 0.47) | 22.27 (5.52 to 57.2) | 0.81 (0.71 to 0.94) | 21.31 (5.37 to 54.62) | 0.83 (0.72 to 0.96) | 0.1 (0.05 to 0.14) | 317.02 (141.51 to 693.85) | 0.8 (0.69 to 0.92) | 309.71 (141.53 to 663.69) | 0.82 (0.71 to 0.94) | 0.18 (-0.03 to 0.38) |
| Western Sub-Saharan Africa | 75.34 (64.05 to 86.53) | 0.85 (0.74 to 0.98) | 73.47 (62.61 to 83.94) | 0.83 (0.72 to 0.96) | -0.08 (-0.15 to -0.01) | 22.9 (5.5 to 65.94) | 0.84 (0.74 to 0.97) | 25.63 (6.31 to 68.48) | 0.86 (0.76 to 0.99) | -0.41 (-0.78 to -0.03) | 309.39 (124 to 769.07) | 0.84 (0.74 to 0.97) | 333.53 (129.48 to 774.76) | 0.85 (0.74 to 0.98) | 0.3 (0.16 to 0.43) |

**Abbreviations**: DALYs, disability-adjusted life-years; EAPCs, estimated annual percentage changes

Below the Rates and Male/Female Ratio column are their estimated values and 95% uncertainty intervals; EAPCs represent the annual percentage change and their 95% confidence intervals in age-standardized rates during 30 years from 1990 to 2019.

**eTable 5.** The age-standardized rates per 100 000 people of incidence, deaths, and DALYs for Parkinson’s disease in 1990 and 2019, with male-to-female ratios and estimated annual percentage changes.

| Location | Age-standardized incidence rates | | | | | Age-standardized death rates | | | | | Age-standardized DALYs rates | | | | |
| --- | --- | --- | --- | --- | --- | --- | --- | --- | --- | --- | --- | --- | --- | --- | --- |
|  | **1990** | | **2019** | | EAPC (%) | 1990 | | 2019 | | EAPC (%) | 1990 | | 2019 | | EAPC (%) |
|  | Rates | Male/Female Ratio | Rates | Male/Female Ratio |  | Rates | Male/Female Ratio | Rates | Male/Female Ratio |  | Rates | Male/Female Ratio | Rates | Male/Female Ratio |  |
| Global | 11.22 (9.87 to 12.68) | 1.52 (1.31 to 1.74) | 13.43 (11.84 to 15.02) | 1.78 (1.55 to 2.05) | 0.77 (0.64 to 0.89) | 4.62 (4.28 to 4.94) | 1.8 (1.55 to 2.07) | 4.79 (4.3 to 5.13) | 1.9 (1.66 to 2.19) | 0.13 (0.08 to 0.19) | 78.09 (72.26 to 84.66) | 1.67 (1.44 to 1.92) | 79.97 (73.25 to 86.6) | 1.79 (1.56 to 2.05) | 0.34 (0.29 to 0.38) |
| Sociodemographic index (SDI) |  |  |  |  |  |  |  |  |  |  |  |  |  |  |  |
| High SDI | 11.31 (10.11 to 12.61) | 1.69 (1.46 to 1.94) | 16.75 (15.11 to 18.31) | 2.44 (2.11 to 2.8) | 1.28 (1.11 to 1.45) | 4.03 (3.7 to 4.19) | 2.06 (1.78 to 2.39) | 4.64 (4.12 to 4.93) | 2.21 (1.92 to 2.53) | -0.03 (-0.11 to 0.04) | 68.3 (63.27 to 73.16) | 1.91 (1.66 to 2.2) | 76.55 (69.65 to 82.39) | 2.02 (1.76 to 2.34) | 0.16 (0.09 to 0.23) |
| High-middle SDI | 12.29 (10.71 to 13.96) | 1.55 (1.35 to 1.78) | 13.36 (11.63 to 15.11) | 1.68 (1.47 to 1.93) | 0.58 (0.32 to 0.84) | 4.83 (4.48 to 5.09) | 1.89 (1.65 to 2.16) | 4.48 (4.04 to 4.82) | 1.99 (1.73 to 2.3) | 0.02 (-0.08 to 0.11) | 81.98 (75.91 to 88.24) | 1.73 (1.5 to 2) | 76.75 (69.4 to 84.08) | 1.86 (1.62 to 2.14) | -0.04 (-0.12 to 0.05) |
| Middle SDI | 11.45 (9.88 to 13.09) | 1.4 (1.21 to 1.6) | 12.86 (11.08 to 14.68) | 1.52 (1.32 to 1.75) | 0.65 (0.44 to 0.87) | 4.91 (4.5 to 5.36) | 1.5 (1.31 to 1.74) | 4.89 (4.34 to 5.37) | 1.72 (1.5 to 1.98) | -0.13 (-0.25 to -0.01) | 82.81 (75.73 to 90.94) | 1.45 (1.26 to 1.67) | 82.22 (74.03 to 90.57) | 1.66 (1.45 to 1.9) | 0.1 (0.01 to 0.19) |
| Low-middle SDI | 9.81 (8.61 to 11.09) | 1.36 (1.18 to 1.56) | 10.78 (9.39 to 12.18) | 1.46 (1.27 to 1.67) | 0.43 (0.34 to 0.51) | 5.04 (4.36 to 6) | 1.55 (1.35 to 1.76) | 5.28 (4.71 to 5.94) | 1.6 (1.4 to 1.84) | 0.42 (0.29 to 0.55) | 82.76 (71.83 to 97.89) | 1.5 (1.31 to 1.75) | 85.44 (76.67 to 95.24) | 1.55 (1.35 to 1.79) | 0.55 (0.46 to 0.63) |
| Low SDI | 8.82 (7.83 to 9.79) | 1.36 (1.18 to 1.57) | 9.32 (8.27 to 10.38) | 1.38 (1.19 to 1.59) | 0.31 (0.22 to 0.4) | 4.72 (3.85 to 6.19) | 1.64 (1.41 to 1.88) | 4.92 (4.29 to 5.81) | 1.55 (1.34 to 1.78) | 0.7 (0.55 to 0.84) | 77.15 (64.71 to 99.4) | 1.59 (1.38 to 1.84) | 78.37 (69.22 to 90.27) | 1.51 (1.32 to 1.74) | 0.14 (0.07 to 0.21) |
| Region |  |  |  |  |  |  |  |  |  |  |  |  |  |  |  |
| Andean Latin America | 10.25 (9.25 to 11.23) | 1.48 (1.29 to 1.7) | 12.58 (11.5 to 13.68) | 1.6 (1.4 to 1.84) | 0.99 (0.76 to 1.23) | 4.93 (4.37 to 5.76) | 1.59 (1.39 to 1.8) | 5.31 (4.36 to 6.29) | 1.62 (1.42 to 1.87) | 0.07 (0 to 0.14) | 80.02 (71.25 to 91.14) | 1.66 (1.45 to 1.91) | 84.06 (70.57 to 97.92) | 1.65 (1.44 to 1.87) | 0.01 (-0.12 to 0.15) |
| Australasia | 13.74 (12.47 to 15.24) | 1.81 (1.57 to 2.1) | 15.23 (13.33 to 17.39) | 2.03 (1.76 to 2.36) | -0.08 (-0.4 to 0.23) | 4.5 (4.13 to 4.71) | 2.33 (2.04 to 2.68) | 4.98 (4.33 to 5.35) | 2.43 (2.11 to 2.77) | -0.04 (-0.17 to 0.09) | 79.74 (73.24 to 86.22) | 2.12 (1.86 to 2.45) | 84.43 (75.44 to 93.15) | 2.26 (1.97 to 2.6) | 1.06 (0.92 to 1.2) |
| Caribbean | 9.72 (8.92 to 10.62) | 1.65 (1.43 to 1.9) | 11.51 (10.51 to 12.54) | 1.72 (1.48 to 1.97) | 0.36 (0.2 to 0.52) | 4.21 (3.86 to 4.48) | 1.69 (1.48 to 1.96) | 4.79 (4.17 to 5.41) | 1.74 (1.5 to 2) | 0.5 (0.44 to 0.57) | 69.22 (64.01 to 75.06) | 1.7 (1.46 to 1.97) | 79.07 (69.72 to 89.1) | 1.76 (1.54 to 2.04) | 0.15 (0.09 to 0.21) |
| Central Asia | 9.86 (8.86 to 10.91) | 1.64 (1.44 to 1.89) | 11.4 (10.47 to 12.42) | 1.59 (1.39 to 1.83) | 0.56 (0.49 to 0.63) | 3.73 (3.28 to 4.63) | 1.83 (1.59 to 2.11) | 5.4 (4.87 to 5.91) | 1.7 (1.48 to 1.96) | 0.17 (0.11 to 0.22) | 63.5 (56.63 to 74.72) | 1.73 (1.51 to 1.99) | 85.45 (77.69 to 93.27) | 1.68 (1.45 to 1.93) | 0.07 (0 to 0.13) |
| Central Europe | 11.33 (10.33 to 12.41) | 1.47 (1.29 to 1.7) | 11.72 (10.83 to 12.62) | 1.55 (1.36 to 1.8) | 0.12 (0.08 to 0.16) | 4.82 (4.51 to 4.99) | 1.74 (1.52 to 2) | 4.76 (4.18 to 5.28) | 1.84 (1.58 to 2.13) | 0.89 (0.58 to 1.21) | 79.23 (74.45 to 84.07) | 1.64 (1.42 to 1.89) | 77.48 (68.76 to 85.7) | 1.76 (1.53 to 2) | 0.67 (0.58 to 0.77) |
| Central Latin America | 9.87 (8.81 to 10.89) | 1.54 (1.33 to 1.78) | 11.11 (9.95 to 12.26) | 1.68 (1.44 to 1.94) | 0.39 (0.33 to 0.45) | 4.28 (3.87 to 4.48) | 1.51 (1.32 to 1.77) | 4.51 (3.91 to 5.13) | 1.7 (1.48 to 1.95) | 0.57 (0.49 to 0.64) | 68.16 (63.22 to 72.35) | 1.55 (1.36 to 1.78) | 73.33 (64.7 to 82.58) | 1.72 (1.51 to 1.99) | -0.04 (-0.2 to 0.12) |
| Central Sub-Saharan Africa | 7.83 (7 to 8.82) | 1.56 (1.35 to 1.79) | 7.96 (7.05 to 8.95) | 1.52 (1.32 to 1.75) | 0.19 (0.1 to 0.27) | 4.32 (3.52 to 5.59) | 1.97 (1.72 to 2.28) | 4.08 (3.32 to 5.32) | 1.82 (1.58 to 2.1) | -0.52 (-0.62 to -0.42) | 72.89 (60.55 to 91.89) | 1.89 (1.65 to 2.2) | 67.95 (56.14 to 84.68) | 1.73 (1.49 to 1.99) | 0.32 (0.25 to 0.38) |
| East Asia | 13.25 (11.25 to 15.41) | 1.42 (1.24 to 1.64) | 15.26 (12.88 to 17.7) | 1.65 (1.43 to 1.9) | 0.9 (0.54 to 1.26) | 5.6 (5.03 to 6.2) | 1.68 (1.47 to 1.94) | 4.64 (3.99 to 5.26) | 2.15 (1.88 to 2.51) | 0.03 (-0.08 to 0.13) | 96.31 (85.95 to 107.18) | 1.53 (1.34 to 1.77) | 83.85 (72.56 to 95.77) | 1.88 (1.63 to 2.16) | -0.8 (-1.14 to -0.46) |
| Eastern Europe | 10.35 (8.74 to 12.05) | 1.54 (1.35 to 1.77) | 10.3 (8.7 to 11.96) | 1.75 (1.51 to 2.02) | 0.01 (-0.05 to 0.07) | 3.79 (3.48 to 4.01) | 1.81 (1.59 to 2.08) | 4.02 (3.58 to 4.4) | 1.76 (1.52 to 2.04) | 0.88 (0.52 to 1.25) | 65.01 (59.17 to 70.54) | 1.68 (1.45 to 1.93) | 67.85 (61.07 to 75.02) | 1.76 (1.52 to 2.01) | 0.2 (0.1 to 0.29) |
| Eastern Sub-Saharan Africa | 7.7 (6.84 to 8.62) | 1.57 (1.37 to 1.81) | 7.7 (6.85 to 8.61) | 1.56 (1.36 to 1.8) | 0.15 (0.03 to 0.26) | 4.08 (3.28 to 5.72) | 1.75 (1.52 to 2.02) | 4.27 (3.61 to 6.42) | 1.7 (1.48 to 1.98) | -0.39 (-0.56 to -0.23) | 69.27 (56.54 to 94.01) | 1.73 (1.51 to 1.96) | 68.74 (59.22 to 96.17) | 1.69 (1.46 to 1.95) | 0.18 (0.13 to 0.23) |
| High-income Asia Pacific | 7.55 (6.41 to 8.7) | 1.58 (1.38 to 1.83) | 9.32 (7.97 to 10.61) | 1.51 (1.32 to 1.75) | 0.52 (0.41 to 0.63) | 3.24 (2.94 to 3.55) | 1.98 (1.74 to 2.28) | 3.51 (2.94 to 3.82) | 1.87 (1.62 to 2.15) | -0.24 (-0.32 to -0.15) | 52.81 (48.66 to 57.22) | 1.79 (1.57 to 2.08) | 58.11 (51.04 to 63.45) | 1.7 (1.48 to 1.94) | -0.16 (-0.38 to 0.05) |
| High-income North America | 11.29 (9.56 to 13.1) | 1.76 (1.53 to 2.02) | 25.14 (21.88 to 28.66) | 3.86 (3.37 to 4.44) | 2.26 (1.74 to 2.79) | 3.79 (3.43 to 3.96) | 1.94 (1.67 to 2.23) | 5.26 (4.72 to 5.55) | 2.35 (2.05 to 2.71) | 0.41 (0.36 to 0.45) | 64.68 (59.66 to 69.65) | 1.86 (1.63 to 2.15) | 84.05 (77.48 to 89.05) | 2.23 (1.94 to 2.58) | 0.53 (0.5 to 0.57) |
| North Africa and Middle East | 10.12 (9.09 to 11.19) | 1.5 (1.3 to 1.7) | 11.4 (10.28 to 12.54) | 1.48 (1.29 to 1.69) | 0.63 (0.49 to 0.77) | 5.18 (4.48 to 6.91) | 1.59 (1.39 to 1.85) | 5.3 (4.57 to 6.89) | 1.51 (1.31 to 1.72) | 0.31 (0.26 to 0.37) | 83.65 (73.26 to 104.56) | 1.51 (1.31 to 1.73) | 84.39 (74.67 to 103.2) | 1.47 (1.28 to 1.69) | 0.86 (0.58 to 1.15) |
| Oceania | 16.21 (14.57 to 18.06) | 1.75 (1.53 to 1.98) | 14.85 (13.33 to 16.54) | 1.64 (1.41 to 1.88) | -0.5 (-0.73 to -0.28) | 8.81 (6.19 to 10.68) | 1.92 (1.67 to 2.22) | 8.05 (5.77 to 9.64) | 1.73 (1.52 to 2) | -0.96 (-1.38 to -0.54) | 146.51 (102.69 to 178.97) | 1.83 (1.59 to 2.11) | 134.2 (94.34 to 160.72) | 1.65 (1.42 to 1.91) | -0.24 (-0.36 to -0.12) |
| South Asia | 9.26 (7.89 to 10.67) | 1.39 (1.21 to 1.6) | 10.07 (8.5 to 11.6) | 1.44 (1.25 to 1.66) | 0.45 (0.3 to 0.61) | 5.31 (4.32 to 6.66) | 1.56 (1.35 to 1.77) | 5.16 (4.44 to 5.98) | 1.52 (1.33 to 1.75) | 0.09 (0.06 to 0.13) | 84.54 (69.83 to 104.73) | 1.52 (1.32 to 1.75) | 82.06 (71.05 to 93.98) | 1.49 (1.31 to 1.74) | -0.11 (-0.25 to 0.03) |
| Southeast Asia | 11.05 (9.84 to 12.31) | 1.31 (1.13 to 1.51) | 11.79 (10.52 to 13.17) | 1.35 (1.18 to 1.56) | 0.29 (0.24 to 0.34) | 4.45 (3.82 to 4.93) | 1.34 (1.16 to 1.55) | 5.04 (4.25 to 5.72) | 1.51 (1.31 to 1.74) | -0.05 (-0.09 to 0) | 76.24 (65.84 to 84.83) | 1.34 (1.17 to 1.53) | 83.38 (71.79 to 93.45) | 1.53 (1.34 to 1.76) | 0.47 (0.35 to 0.59) |
| Southern Latin America | 12.11 (11.07 to 13.33) | 2.06 (1.8 to 2.37) | 12.21 (11.03 to 13.45) | 2.25 (1.96 to 2.59) | 0.36 (0.19 to 0.52) | 5.18 (4.81 to 5.46) | 2.3 (1.98 to 2.63) | 5.41 (4.9 to 5.74) | 2.37 (2.07 to 2.72) | 0.45 (0.37 to 0.53) | 82.55 (77.2 to 87.98) | 2.21 (1.92 to 2.52) | 83.72 (77.06 to 89.63) | 2.35 (2.05 to 2.74) | -0.03 (-0.06 to -0.01) |
| Southern Sub-Saharan Africa | 7.95 (6.85 to 9.02) | 1.61 (1.41 to 1.88) | 9.24 (7.94 to 10.58) | 1.58 (1.38 to 1.84) | 0.6 (0.54 to 0.66) | 3.11 (2.78 to 3.47) | 1.62 (1.4 to 1.86) | 4.11 (3.7 to 4.44) | 1.64 (1.43 to 1.88) | -0.05 (-0.12 to 0.03) | 49.24 (44.57 to 54.74) | 1.69 (1.48 to 1.95) | 62.47 (56.83 to 67.42) | 1.71 (1.5 to 1.98) | -0.06 (-0.24 to 0.11) |
| Tropical Latin America | 9.63 (8.09 to 11.15) | 1.42 (1.24 to 1.63) | 10.83 (9.13 to 12.58) | 1.44 (1.26 to 1.63) | 0.37 (0.29 to 0.45) | 4.52 (4.07 to 4.76) | 1.48 (1.3 to 1.71) | 4.44 (3.91 to 4.77) | 1.55 (1.36 to 1.81) | 1.24 (1.1 to 1.37) | 71.24 (65.98 to 75.91) | 1.56 (1.36 to 1.79) | 71.88 (65.26 to 77.85) | 1.61 (1.4 to 1.86) | 0.77 (0.52 to 1.02) |
| Western Europe | 13.66 (12.42 to 14.94) | 1.6 (1.4 to 1.86) | 14.61 (13.03 to 16.05) | 1.65 (1.44 to 1.92) | 0.54 (0.41 to 0.66) | 4.47 (4.14 to 4.63) | 2.25 (1.96 to 2.59) | 4.82 (4.3 to 5.12) | 2.29 (1.99 to 2.63) | 0.16 (0.07 to 0.24) | 76.5 (70.72 to 82.54) | 1.98 (1.71 to 2.28) | 80.02 (72.6 to 87.02) | 2.03 (1.77 to 2.33) | 0.46 (0.39 to 0.53) |
| Western Sub-Saharan Africa | 9.74 (8.64 to 10.8) | 1.19 (1.04 to 1.36) | 10.76 (9.67 to 11.83) | 1.2 (1.04 to 1.36) | 0.48 (0.39 to 0.58) | 5.1 (4.4 to 6.02) | 1.22 (1.07 to 1.41) | 5.97 (5.11 to 6.7) | 1.25 (1.09 to 1.41) | 0.2 (0.06 to 0.33) | 76.63 (66.91 to 89.14) | 1.27 (1.11 to 1.47) | 87.3 (76.25 to 97.98) | 1.28 (1.12 to 1.48) | 0.08 (0.03 to 0.14) |

**Abbreviations**: DALYs, disability-adjusted life-years; EAPCs, estimated annual percentage changes

Below the Rates and Male/Female Ratio column are their estimated values and 95% uncertainty intervals; EAPCs represent the annual percentage change and their 95% confidence intervals in age-standardized rates during 30 years from 1990 to 2019.

**eTable 6.** Cross-country slope index of inequality and concentration index according to years lived with disability, and years of life lost, in 1990 and 2019 for Alzheimer’s disease and related dementias and Parkinson’s disease among both sexes, males, and females.

| Metric | Disease | Sex | Health inequality index | Year | Estimate | 95% CI |
| --- | --- | --- | --- | --- | --- | --- |
| Years Lived with Disability (YLDs) | Alzheimer's disease and related dementias | Both | Slope index of inequality | 1990 | 79.6 | 66.2 to 92.6 |
|  |  |  |  | 2019 | 175.6 | 156.1 to 195.1 |
|  |  |  | Concentration index | 1990 | 38.6 | 35.5 to 41.7 |
|  |  |  |  | 2019 | 41.2 | 37.3 to 45.1 |
|  |  | Male | Slope index of inequality | 1990 | 48.5 | 40.9 to 56.1 |
|  |  |  |  | 2019 | 118.9 | 105.9 to 132.0 |
|  |  |  | Concentration index | 1990 | 30.9 | 28.4 to 33.5 |
|  |  |  |  | 2019 | 36.6 | 33.1 to 40.2 |
|  |  | Female | Slope index of inequality | 1990 | 109.6 | 91.0 to 128.2 |
|  |  |  |  | 2019 | 229.6 | 203.3 to 256.0 |
|  |  |  | Concentration index | 1990 | 42.0 | 38.7 to 45.4 |
|  |  |  |  | 2019 | 43.6 | 39.5 to 47.8 |
|  | Parkinson's disease | Both | Slope index of inequality | 1990 | 12.7 | 10.9 to 14.5 |
|  |  |  |  | 2019 | 26.4 | 23.7 to 29.0 |
|  |  |  | Concentration index | 1990 | 30.5 | 27.4 to 33.7 |
|  |  |  |  | 2019 | 34.9 | 30.4 to 39.4 |
|  |  | Male | Slope index of inequality | 1990 | 12.3 | 10.5 to 14.1 |
|  |  |  |  | 2019 | 27.6 | 25.0 to 30.3 |
|  |  |  | Concentration index | 1990 | 27.7 | 24.7 to 30.7 |
|  |  |  |  | 2019 | 34.6 | 29.9 to 39.3 |
|  |  | Female | Slope index of inequality | 1990 | 12.8 | 10.9 to 14.7 |
|  |  |  |  | 2019 | 24.4 | 22.0 to 26.8 |
|  |  |  | Concentration index | 1990 | 33.6 | 30.2 to 36.9 |
|  |  |  |  | 2019 | 35.4 | 31.0 to 39.7 |
| Years of Life Lost (YLLs) | Alzheimer's disease and related dementias | Both | Slope index of inequality | 1990 | 174.1 | 147.1 to 201.1 |
|  |  |  |  | 2019 | 382.3 | 339.3 to 425.3 |
|  |  |  | Concentration index | 1990 | 31.7 | 21.4 to 42.1 |
|  |  |  |  | 2019 | 35.2 | 22.9 to 47.5 |
|  |  | Male | Slope index of inequality | 1990 | 108.6 | 91.0 to 126.1 |
|  |  |  |  | 2019 | 266.3 | 236.9 to 295.7 |
|  |  |  | Concentration index | 1990 | 24.5 | 16.0 to 32.9 |
|  |  |  |  | 2019 | 30.8 | 19.6 to 42.0 |
|  |  | Female | Slope index of inequality | 1990 | 233.0 | 196.3 to 269.7 |
|  |  |  |  | 2019 | 493.3 | 439.6 to 547.0 |
|  |  |  | Concentration index | 1990 | 35.4 | 23.7 to 47.0 |
|  |  |  |  | 2019 | 37.7 | 24.7 to 50.7 |
|  | Parkinson's disease | Both | Slope index of inequality | 1990 | 52.9 | 43.5 to 62.4 |
|  |  |  |  | 2019 | 104.7 | 92.7 to 116.7 |
|  |  |  | Concentration index | 1990 | 20.4 | 19.6 to 21.2 |
|  |  |  |  | 2019 | 27.6 | 26.4 to 28.8 |
|  |  | Male | Slope index of inequality | 1990 | 56.4 | 46.0 to 67.2 |
|  |  |  |  | 2019 | 124.3 | 109.6 to 139.0 |
|  |  |  | Concentration index | 1990 | 18.8 | 17.8 to 19.8 |
|  |  |  |  | 2019 | 28.7 | 27.0 to 30.3 |
|  |  | Female | Slope index of inequality | 1990 | 48.1 | 39.4 to 56.8 |
|  |  |  |  | 2019 | 83.0 | 73.0 to 93.1 |
|  |  |  | Concentration index | 1990 | 22.6 | 21.4 to 23.7 |
|  |  |  |  | 2019 | 26.2 | 24.7 to 27.7 |

**Abbreviations:** CI, confidence interval.

**eFigure 1.** The global maps among males for age-standardized rates per 100,000 people of incidence, deaths, and DALYs in 2019 for Alzheimer’s disease and related dementias and Parkinson’s disease.

**
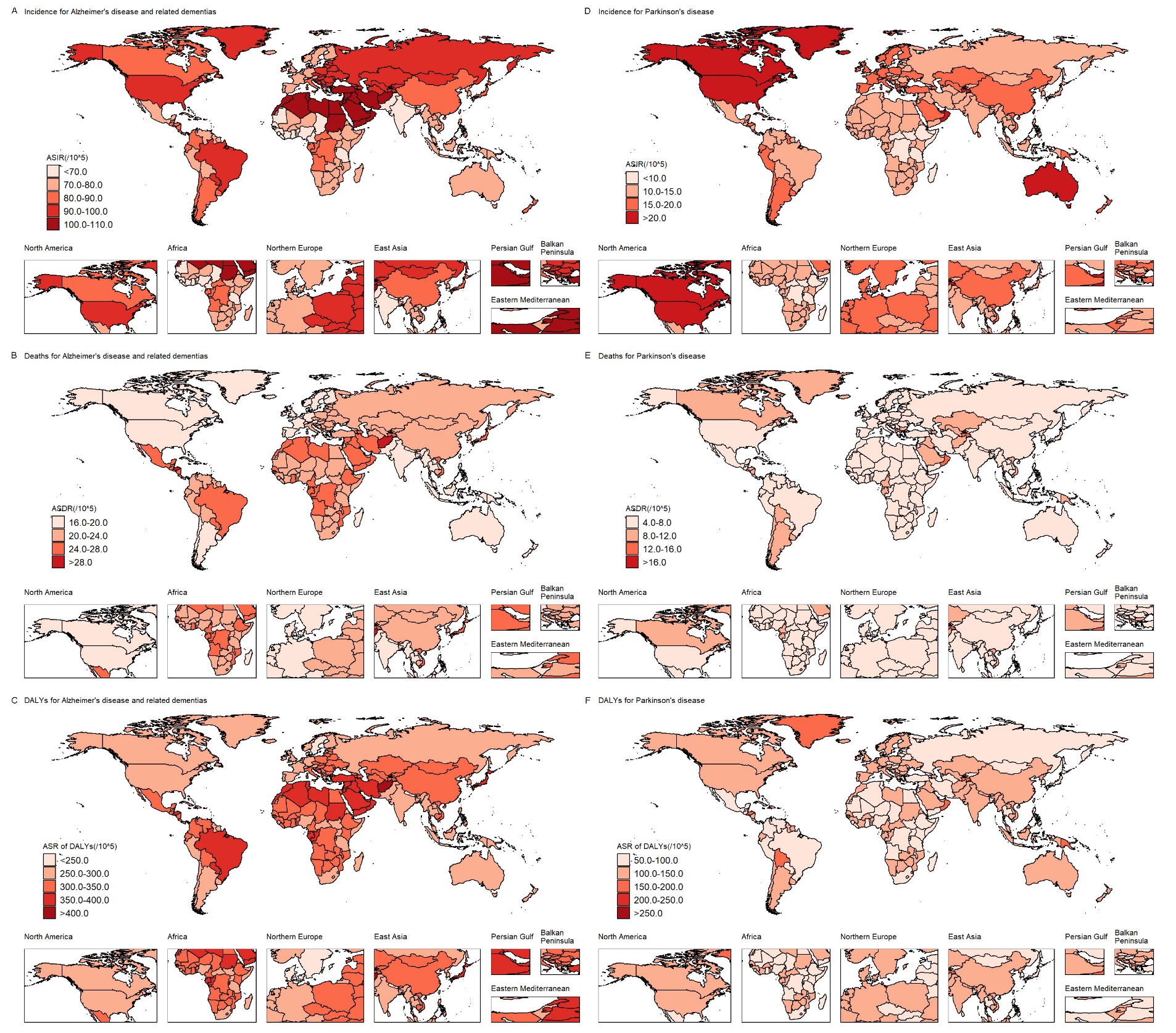
**

**Abbreviations**: DALYs, disability-adjusted life-years;

Figures A, B, and C represent age-standardized rates of incidence, deaths, and disability-adjusted life years for Alzheimer’s disease and related dementias, and Figures D, E, and F represent age-standardized rates of incidence, deaths, and disability-adjusted life years for Parkinson’s disease.

Darker colors in the figures indicate higher age-standardized rates.

**eFigure 2.** The global maps among females for age-standardized rates per 100,000 people of incidence, deaths, and DALYs in 2019 for Alzheimer’s disease and related dementias and Parkinson’s disease.

**
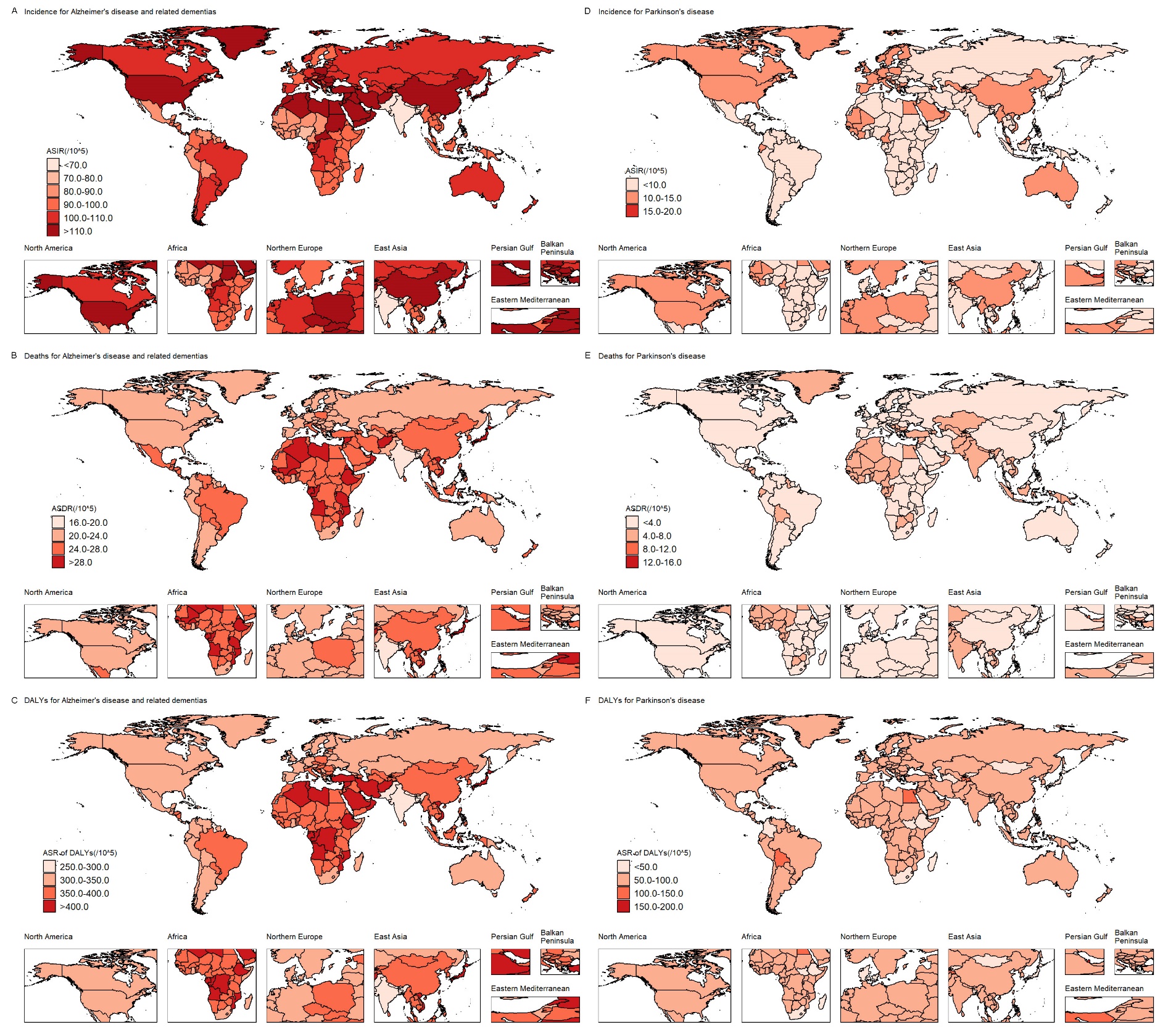
**

**Abbreviations**: DALYs, disability-adjusted life-years;

Figures A, B, and C represent age-standardized rates of incidence, deaths, and disability-adjusted life years for Alzheimer’s disease and related dementias, and Figures D, E, and F represent age-standardized rates of incidence, deaths, and disability-adjusted life years for Parkinson’s disease.

Darker colors in the figures indicate higher age-standardized rates.

**eFigure 3.** Association among age-standardized incidence rates and sociodemographic index for Alzheimer’s disease and related dementias and Parkinson’s disease in males and females.


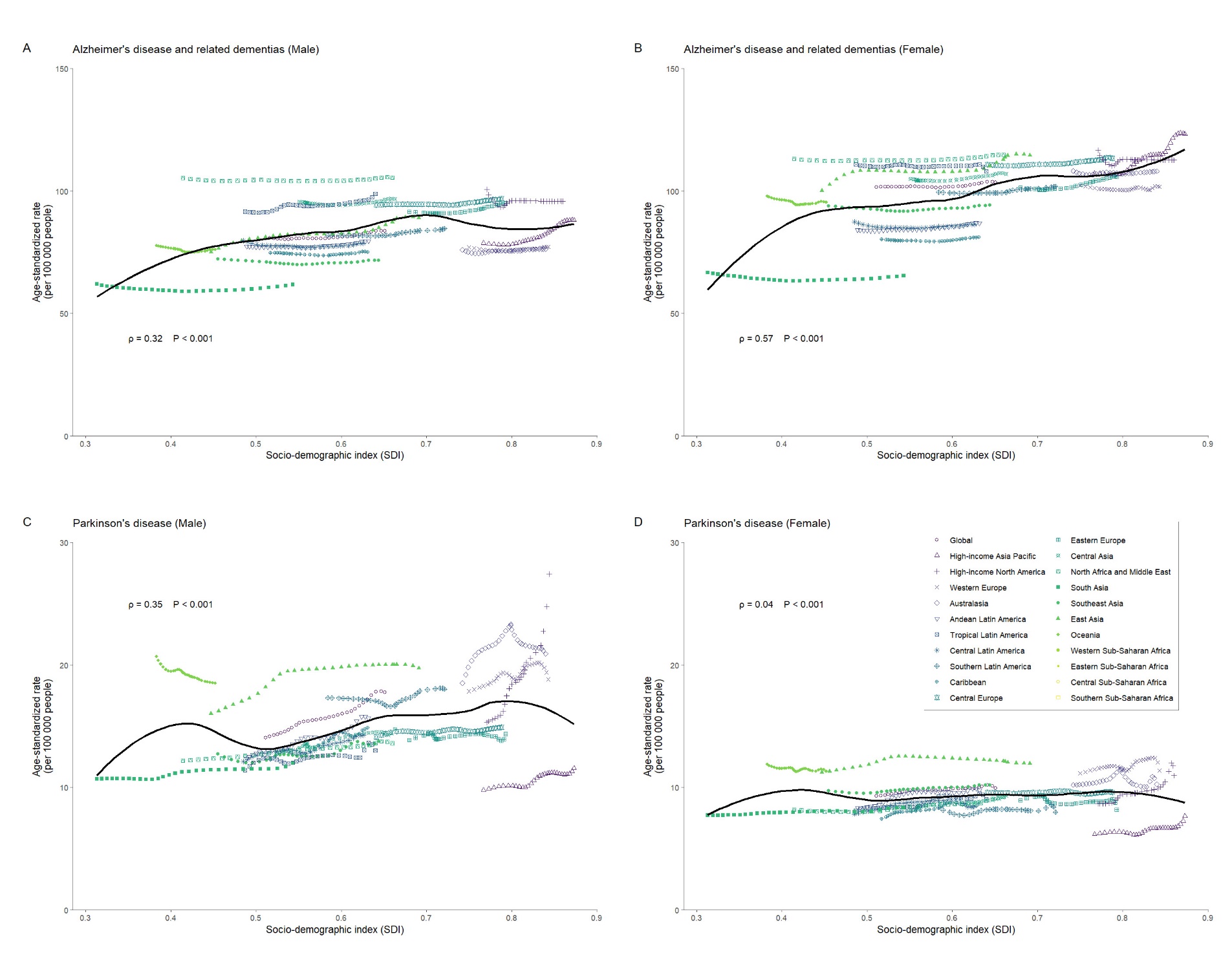


The black solid curves coordinate with the overall trend in age-standardized incidence rates, with Pearson correlation coefficients (ρ index) and *P* values indicating the magnitude and statistical significance of the correlation.

**eFigure 4.** Association between age-standardized incidence rates, sociodemographic index and estimated annual percentage changes, individually, for Alzheimer’s disease and related dementias among males.


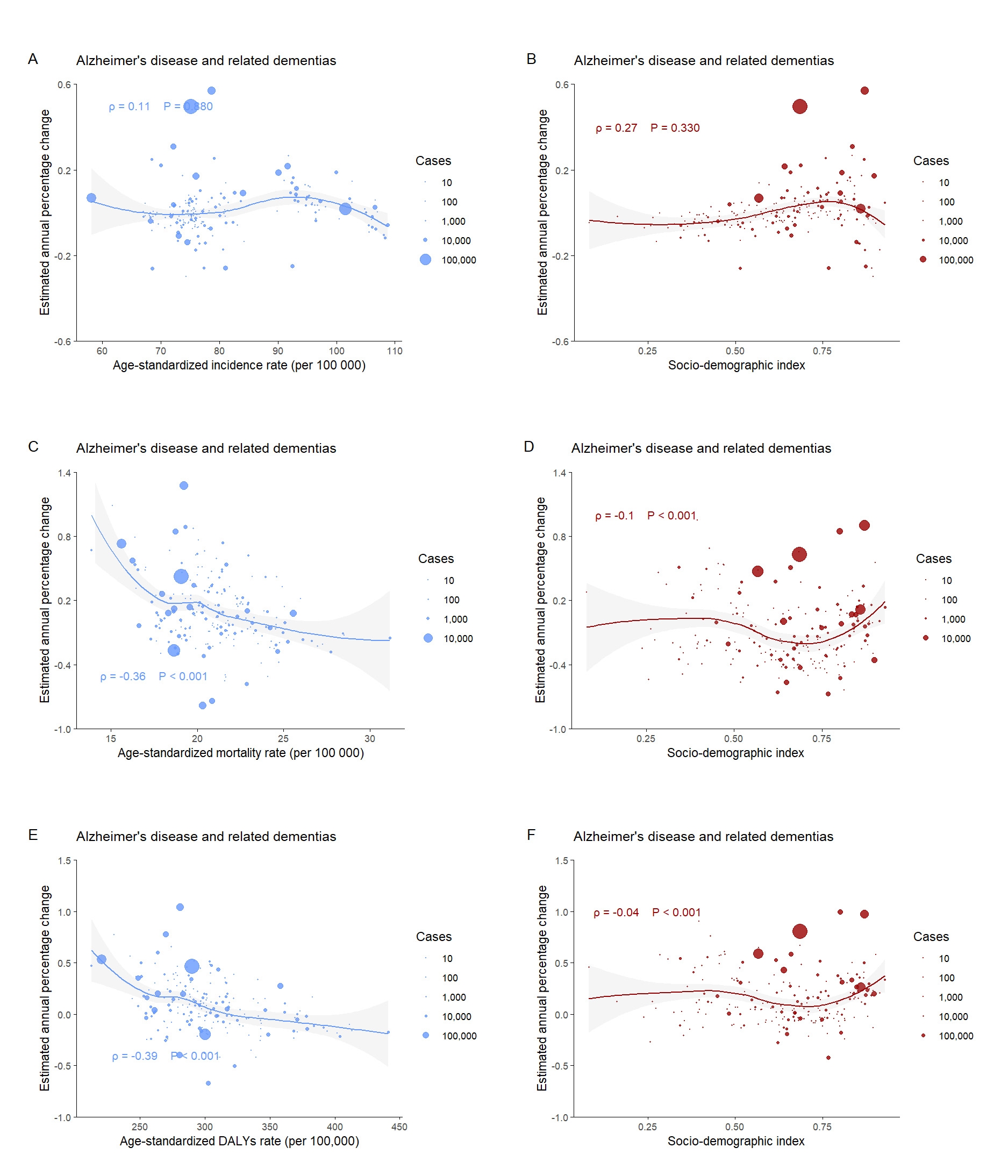


Circles represent the cases of absolute incidence, deaths, and DALYs, the larger the circle the greater the number of cases. EAPCs are 30-year trends in age-standardized incidence, deaths, and disability-adjusted life year rates per 100 000 people. Pearson correlation coefficients (ρ index) and *P* values indicate the magnitude and statistical significance of the correlation. Figures A and B denote age-standardized rates, socio-demographic index and estimated annual percentage changes for incidence, individually; Figures C and D denote age-standardized rates, socio-demographic index and estimated annual percentage changes for deaths, individually; Figures E and F denote age-standardized rates, socio-demographic index and estimated annual percentage changes for DALYs, individually.

**eFigure 5.** Association between age-standardized incidence rates, sociodemographic index and estimated annual percentage changes, individually, for Alzheimer’s disease and related dementias among females.


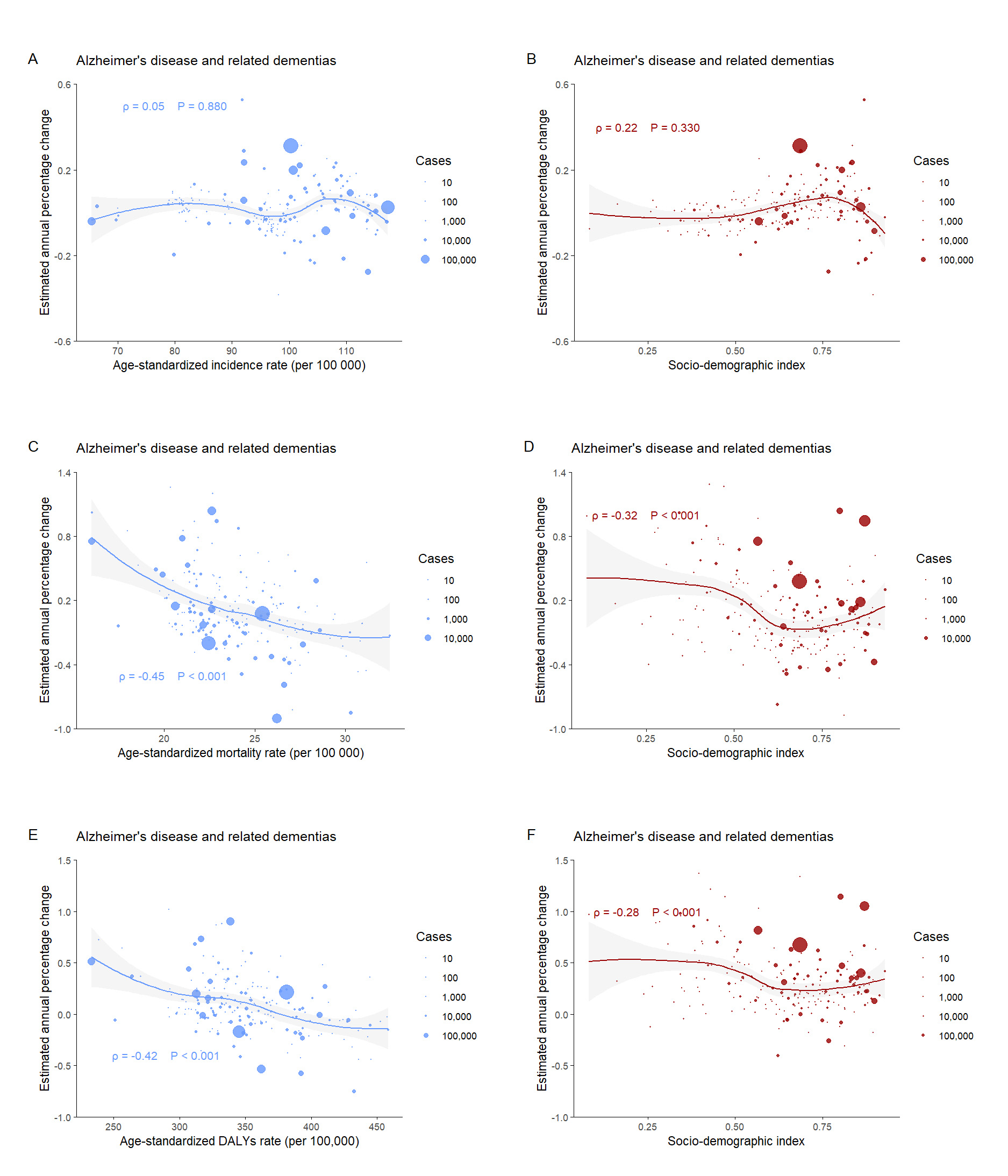


Circles represent the cases of absolute incidence, deaths, and DALYs, the larger the circle the greater the number of cases. EAPCs are 30-year trends in age-standardized incidence, deaths, and disability-adjusted life year rates per 100 000 people. Pearson correlation coefficients (ρ index) and *P* values indicate the magnitude and statistical significance of the correlation. Figures A and B denote age-standardized rates, socio-demographic index and estimated annual percentage changes for incidence, individually; Figures C and D denote age-standardized rates, socio-demographic index and estimated annual percentage changes for deaths, individually; Figures E and F denote age-standardized rates, socio-demographic index and estimated annual percentage changes for DALYs, individually.

**eFigure 6.** Association between age-standardized incidence rates, sociodemographic index and estimated annual percentage changes, individually, for Parkinson’s disease among males.


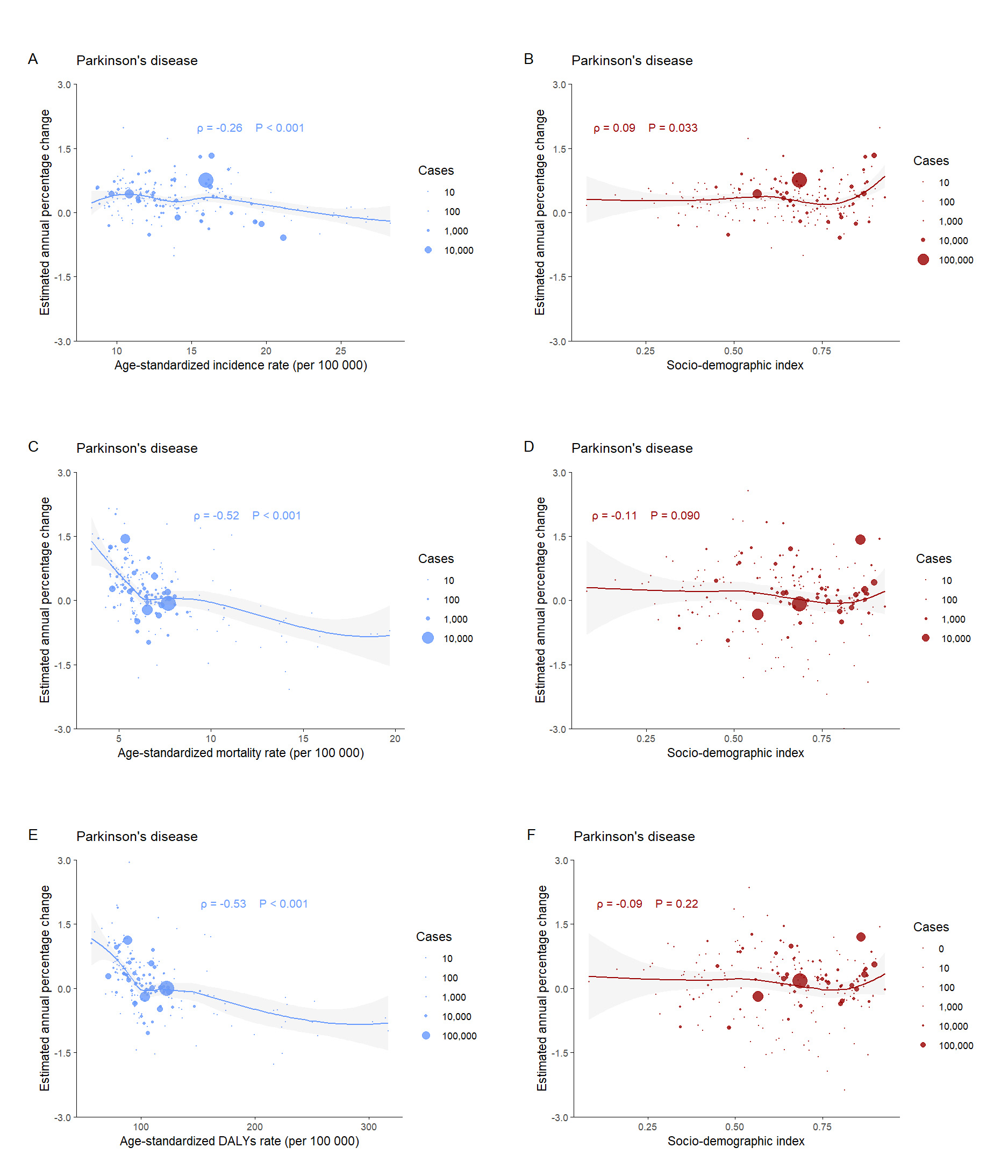


Circles represent the cases of absolute incidence, deaths, and DALYs, the larger the circle the greater the number of cases. EAPCs are 30-year trends in age-standardized incidence, deaths, and disability-adjusted life year rates per 100 000 people. Pearson correlation coefficients (ρ index) and *P* values indicate the magnitude and statistical significance of the correlation. Figures A and B denote age-standardized rates, socio-demographic index and estimated annual percentage changes for incidence, individually; Figures C and D denote age-standardized rates, socio-demographic index and estimated annual percentage changes for deaths, individually; Figures E and F denote age-standardized rates, socio-demographic index and estimated annual percentage changes for DALYs, individually.

**eFigure 7.** Association between age-standardized incidence rates, sociodemographic index and estimated annual percentage changes, individually, for Parkinson’s disease among females.


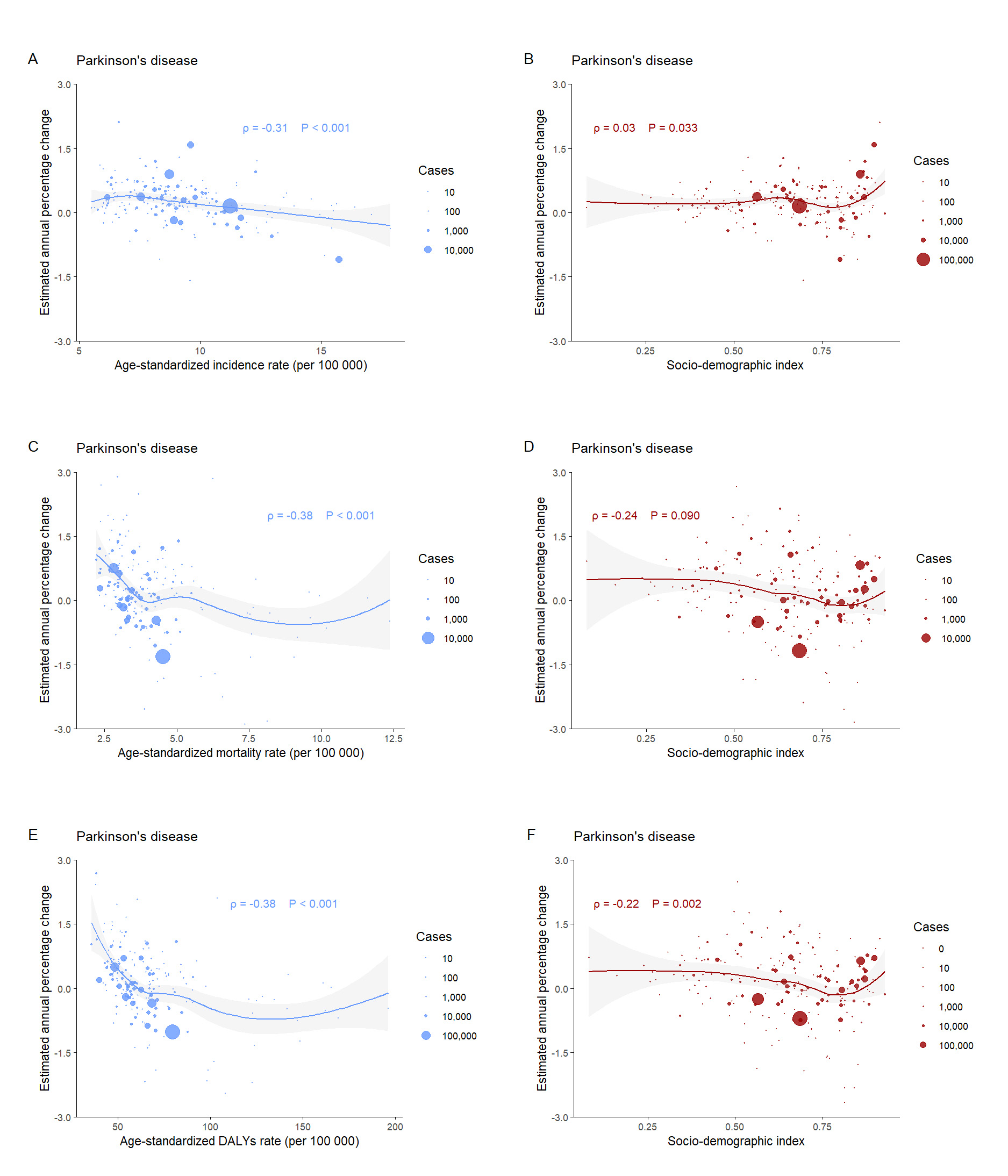


Circles represent the cases of absolute incidence, deaths, and DALYs, the larger the circle the greater the number of cases. EAPCs are 30-year trends in age-standardized incidence, deaths, and disability-adjusted life year rates per 100 000 people. Pearson correlation coefficients (ρ index) and *P* values indicate the magnitude and statistical significance of the correlation. Figures A and B denote age-standardized rates, socio-demographic index and estimated annual percentage changes for incidence, individually; Figures C and D denote age-standardized rates, socio-demographic index and estimated annual percentage changes for deaths, individually; Figures E and F denote age-standardized rates, socio-demographic index and estimated annual percentage changes for DALYs, individually.

**eFigure 8.** Cross-country slope index of inequality and concentration index in 1990 and 2019 for Alzheimer’s disease and related dementias and Parkinson’s disease among males.


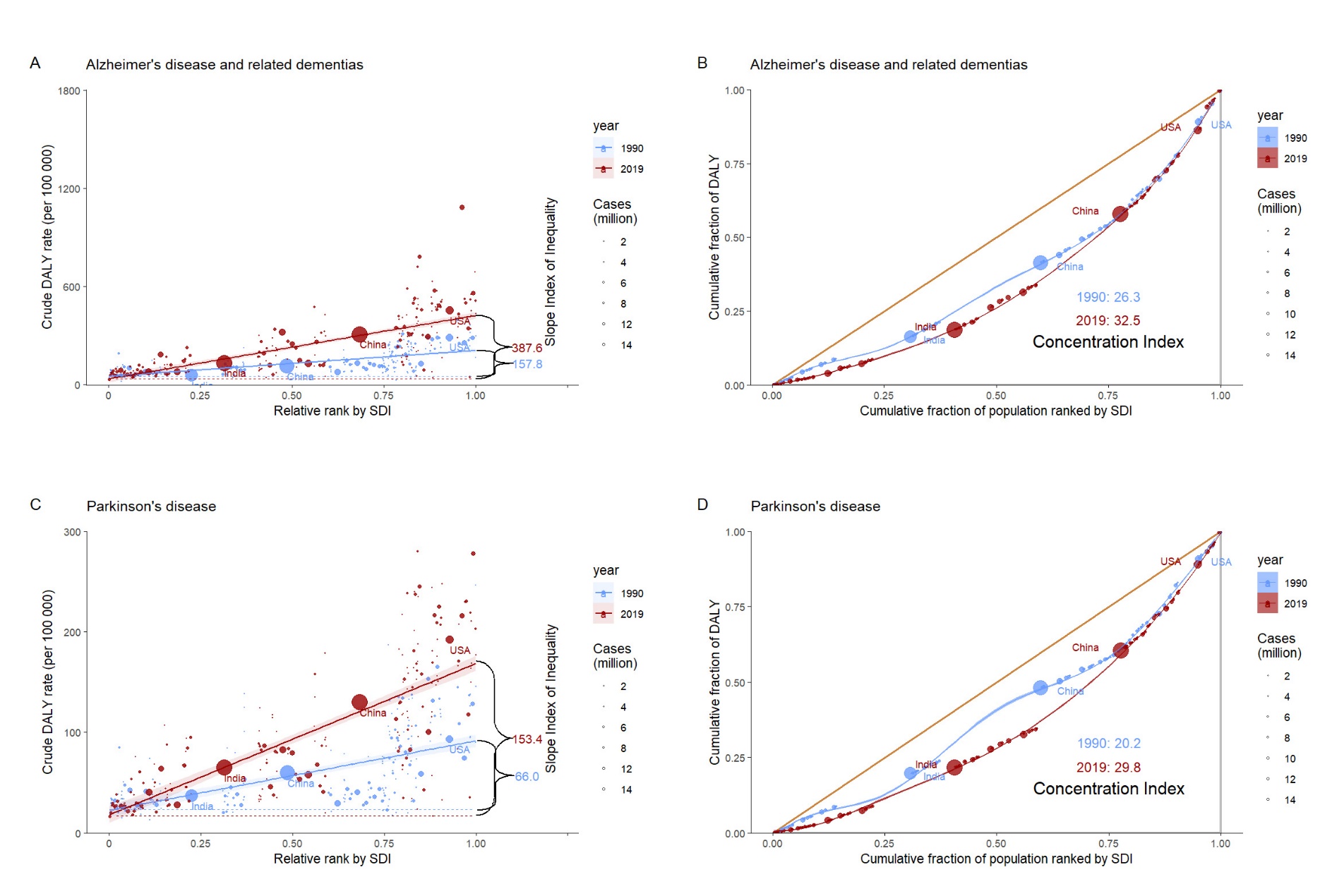


Circles represent the cases of absolute incidence, deaths, and DALYs, the larger the circle the greater the number of cases. Red lines and circles represent data for 2019, while the blue ones indicate data for 1990. Figures A and C denote the slope index of inequality for AD and PD, respectively, and Figures B and D denote the concentration index for AD and PD, respectively.

**eFigure 9.** Cross-country slope index of inequality and concentration index in 1990 and 2019 for Alzheimer’s disease and related dementias and Parkinson’s disease among females.


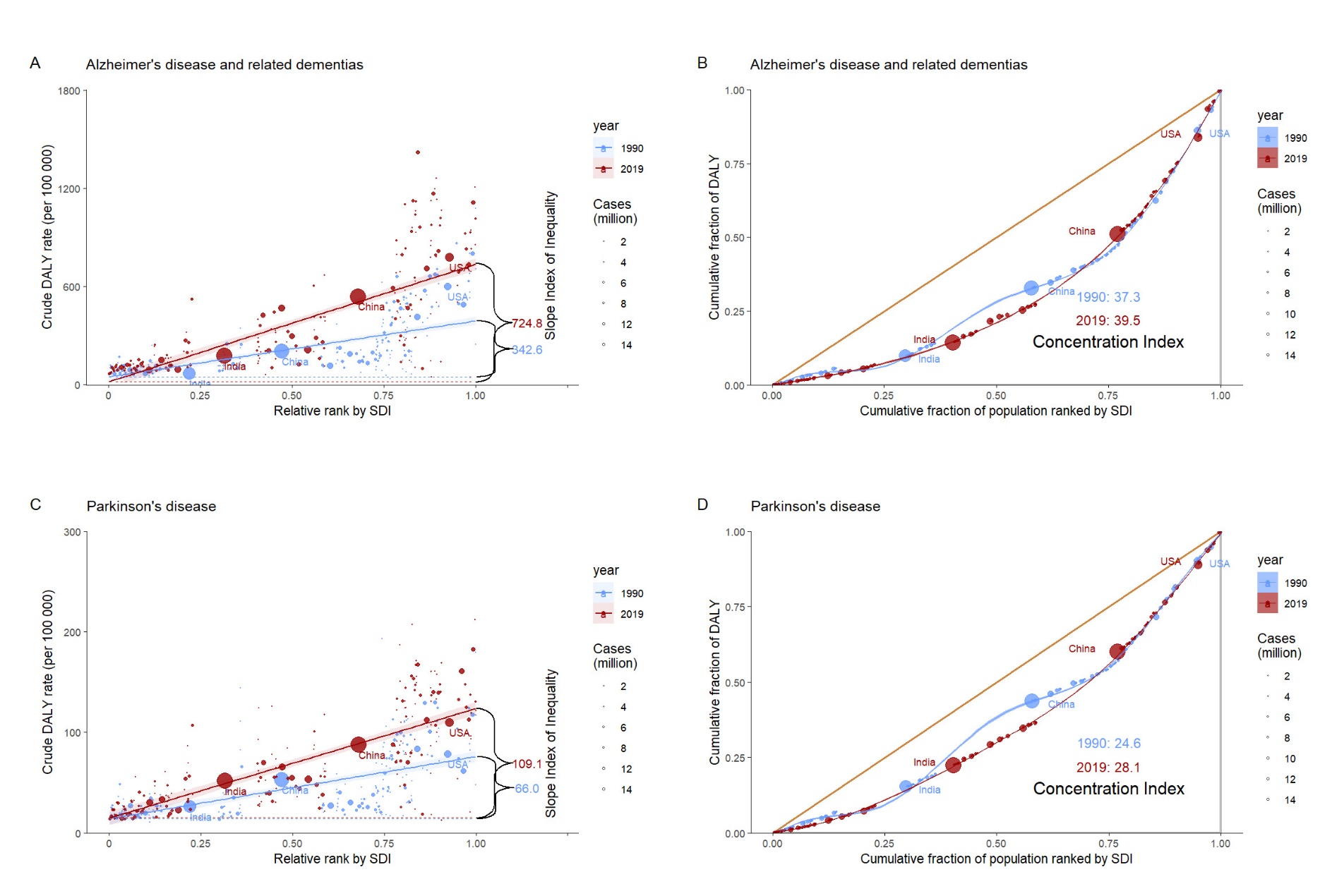


Circles represent the cases of absolute incidence, deaths, and DALYs, the larger the circle the greater the number of cases. Red lines and circles represent data for 2019, while the blue ones indicate data for 1990. Figures A and C denote the slope index of inequality for AD and PD, respectively, and Figures B and D denote the concentration index for AD and PD, respectively.
